# Meat intake, cooking methods, dietary carcinogens, and colorectal cancer risk: findings from the Colorectal Cancer Family Registry

**DOI:** 10.1002/cam4.461

**Published:** 2015-04-07

**Authors:** Amit D Joshi, Andre Kim, Juan Pablo Lewinger, Cornelia M Ulrich, John D Potter, Michelle Cotterchio, Loic Le Marchand, Mariana C Stern

**Affiliations:** 1 Department of Preventive Medicine, Keck School of Medicine of USC, University of Southern CaliforniaLos Angeles, California, 90089; 2 National Center for Tumor Diseases and German Cancer Research CenterHeidelberg, Germany; 3 Public Health Sciences Division, Fred Hutchinson Cancer Research CenterSeattle, Washington, 98109; 4 Centre for Public Health Research, Massey UniversityWellington, New Zealand; 5 Cancer Care OntarioToronto, Ontario, Canada; 6 University of Hawaii Cancer CenterHonolulu, Hawaii, 96822

**Keywords:** Colorectal cancer, heterocyclic amines, mismatch repair proficiency, pan-fried meat, red meat

## Abstract

Diets high in red meat and processed meats are established colorectal cancer (CRC) risk factors. However, it is still not well understood what explains this association. We conducted comprehensive analyses of CRC risk and red meat and poultry intakes, taking into account cooking methods, level of doneness, estimated intakes of heterocyclic amines (HCAs) that accumulate during meat cooking, tumor location, and tumor mismatch repair proficiency (MMR) status. We analyzed food frequency and portion size data including a meat cooking module for 3364 CRC cases, 1806 unaffected siblings, 136 unaffected spouses, and 1620 unaffected population-based controls, recruited into the CRC Family Registry. Odds ratios (OR) and 95% confidence intervals (CI) for nutrient density variables were estimated using generalized estimating equations. We found no evidence of an association between total nonprocessed red meat or total processed meat and CRC risk. Our main finding was a positive association with CRC for pan-fried beefsteak (*P*_trend_ < 0.001), which was stronger among MMR deficient cases (heterogeneity *P* = 0.059). Other worth noting associations, of borderline statistical significance after multiple testing correction, were a positive association between diets high in oven-broiled short ribs or spareribs and CRC risk (*P*_trend_ = 0.002), which was also stronger among MMR-deficient cases, and an inverse association with grilled hamburgers (*P*_trend_ = 0.002). Our results support the role of specific meat types and cooking practices as possible sources of human carcinogens relevant for CRC risk.

## Introduction

Colorectal cancer (CRC) is the second most common cancer in the Western world, with varying incidence rates among different ethnic/racial groups. An expert panel from the World Cancer Research Fund and American Institute for Cancer Research reported convincing evidence that red meat (“beef, pork, lamb and goat from domesticated animals”) and processed meat (“preserved by smoking, curing, or salting, or addition of chemical preservatives”) increase the risk of CRC. The data were insufficient to make any conclusions about the role of poultry in cancer risk 1.

Several mechanisms have been proposed to explain the link between meat consumption and CRC risk. Diets high in meats may displace cancer-protective factors, such as fruits, vegetables, dietary fibers, and plant-based sources of anticancer bioactives, such as flavonoids and isothiocyanates 2. Meats are rich sources of fatty acids, which have been postulated as possible CRC risk factors 2. Carcinogenic *N*-nitroso compounds can be found in many processed meats and can be endogenously formed after ingesting red meat in the human intestines with the help of the colonic flora 3. Moreover, the process of cooking meats at high temperatures leads to the formation of other chemical carcinogens; specifically, heterocyclic amines (HCAs) 4 and polycyclic aromatic hydrocarbons (PAHs) 5. Finally, red meat is also a source of heme iron, which may promote oxidative damage 6; heme also participates in the above mentioned process of endogenous *N*-nitrosation in the bowel 7. A meta-analysis of prospective cohort studies reported evidence for a positive association between heme intake and CRC risk 8.

The amount and types of carcinogens that accumulate in cooked meats are dependent on the meat type, the cooking methods, and the temperature and the duration of cooking 9. Pan-frying, grilling, and barbecuing have been shown to produce high levels of mutagens 10, with pan-frying yielding higher mutagenic activity than grilling at a similar temperature 11. Human-controlled feeding studies have shown that the mutagenic activity in urine increases after consumption of cooked meats 12,13. PAHs form through pyrolysis of fats in meats when cooked over open flames, and thus is mostly associated with barbecued (BBQ) meats 14. Feeding studies and cross-sectional studies support a link between charbroiled food consumption and formation of PAH adducts in circulating lymphocytes 15–17. HCAs form through the Maillard reaction involving creatine, amino acids, and sugars, as the cooking temperature increases 18. Human feeding studies reported detectable HCA adducts in the colon and HCA metabolites in urine after intake of dietary relevant doses and cross-sectional studies confirm that HCAs can form through regular diet in the United States and Europe 19–21.

Whereas many studies have studied the role of diets high in red meat and CRC risk, fewer had data to investigate this issue in detail, taking into account cooking methods and/or carcinogen intake 22–32. In this study, we conducted comprehensive analyses of red meat and poultry consumption, taking into account cooking practices, estimated levels of HCAs, tumor location, and tumor molecular characteristics. We conducted these analyses using data from the Colorectal Cancer Family Registry (Colon CFR).

## Methods

### Study subjects

The characteristics of the Colon CFR have been previously described 33. In this case–control study, we included incident cases with CRC (affected probands) who were recruited through population-based registries in three of the six centers of the Colon CFR: Cancer Care Ontario (CCO), University of Hawaii (UH), and the University of Southern California (USC) Consortium, which include the Arizona Cancer Center, Dartmouth College, the University of Colorado, the University of Minnesota, the University of North Carolina, and the University of Southern California. Individuals recruited at these three Colon CFR centers completed a quantitative food frequency questionnaire (FFQ) that included a meat cooking module, and therefore had suitable information for this study. We included, from these centers, all affected probands (cases) who had FFQ data available and who completed the interview ≤5 years after diagnosis, for a total of 3486 cases (1887 from CCO, 1153 from USC, 446 from UH). As controls we included unaffected siblings from the families of the probands (*N* = 1812; 977 from CCO, 499 from USC, 336 from UH), unaffected spouses of the probands (*N* = 141 from UH), and unaffected population-based controls (*N* = 1620 from CCO). These controls were included if they were unaffected at the time of diagnosis of the proband (for sibling and spouse controls) or unaffected at the time of recruitment (population-based controls). Details on ascertainment and eligibility criteria used by the CFR were summarized by Newcomb and colleagues 33. All participants signed a written informed consent approved by the Institutional Review Board of each institution, completed an in-person risk factor questionnaire (RFQ), a quantitative FFQ, and a subset of them donated blood or buccal samples. All cases were pathologically confirmed. Tumor tissue blocks were available from approximately 70% of the cases, providing data on tumor characteristics, such as mismatch repair (MMR) status.

### Data collection

All participants answered a baseline RFQ, which was designed to be used by all colon CFR sites 33 and includes questions covering established and suspected risk factors for CRC, including medical history and medication use, reproductive history (for female participants), family history, physical activity, demographics, alcohol and tobacco use, and limited dietary factors. The method of administration of the questionnaire varied by center: face-to-face interview, Computer-Assisted Telephone Interview (CATI), or mail for self-administration. Manuals for administration were developed for further standardization. In addition to the risk factor core questionnaire, the Hawaii, Ontario, and USC registry centers also administered a detailed quantitative FFQ developed at the University of Hawaii Cancer Center (UHCC) 34, which included a meat/fish cooking module specifically developed to address the role of meat carcinogens. All questions were asked in reference to two years before the cancer diagnosis. For a subset of cases (*N* = 1119), data were available either for microsatellite instability (MSI) status or for immunohistochemistry (IHC) phenotype or both. We classified CRC patients as MMR-deficient if there was evidence of MSI-high status or abnormal IHC phenotype 35. Patients with MSI-stable/low status and normal IHC phenotypes were defined as MMR-proficient. If either MSI or IHC status was missing, the determination was made on the basis of the available phenotype. Cases with both MSI and IHC data missing were not assigned MMR status. We were also able to classify patients into sporadic or familial cancer based on Amsterdam criteria.

### Assessment of meat intake and meat cooking practices

A validated FFQ was mailed to participants who had answered the RFQ and was returned after completion by 79.6% of study participants in three centers (CCO, USC, and UH). This FFQ obtained data on the usual consumption of more than 200 food items and more than 100 nutrients, including data on portion sizes and frequency of intake of many relevant meat items, including beef, poultry, lamb, pork, and fish as separate items and as part of mixed dishes; processed meats (meats that have been treated with preservatives such as nitrites or nitrites, or treated by curing, smoking, salting, etc.); and fish and processed fish 34. This FFQ has been validated in a random sample of 2000 Hawaii and California residents using 24-h recalls 34. These data (portion size, frequency, and total intake) were used to derive amount (g/day) of each of the individual meat types and summary variables, including: total nonprocessed red meat (beef, pork, veal, lamb, or game); total poultry (chicken, turkey); total processed red meat (bacon, hot dogs, luncheon meats, sausages); total processed poultry. We also examined some of the individual meat types, including: beef, pork, organ meat, sausages, and lunchmeats. In addition, this FFQ contains a meat/fish cooking module which includes questions on frequency of intake of eight meat items (beefsteak, hamburger, chicken, spam or ham, bacon, sausage, short ribs or spareribs, and fish), cooked by either of three different methods (pan-fried, oven-broiled, and grilled or BBQ), and with three levels of doneness (light, medium, dark). These variables were used to derive meat type/cooking method frequency (servings/day) variables for each combination of meat type and cooking method. We obtained estimated intake levels of individual HCAs (3,8-dimethyl-3H-imidazo[4,5-f]quinoxalin-2-amine, MeIQx; 3,4,8-trimethyl-3H-imidazo[4,5-f]quinoxalin-2-amine, DiMeIQx; and 2-amino-1-methyl-6-phenylimidazo(4,5-b)pyridine, PhIP) using the CHARRED database 36, as previously described 24. All intake variables were energy-adjusted using the nutrient density method (in g/1000 kcal/day) and were categorized into quintiles for individual meat types and meat-derived carcinogens and quartiles for meat cooking variables. We also considered the use of two types of marinades, which were asked separately for red meat or chicken. Two types of marinades were considered: teriyaki sauce or shoyu sauce (soy sauce) and BBQ sauce. We created categories of marinated red meat or poultry consumption (never, <1 per month, >1 per month).

### Data analyses

In our analyses, we excluded participants with extreme daily caloric intake levels (<600 or >6000 kcal/day). 203 observations were excluded: 136 cases (3.9%) and 67 controls (1.9%). We also excluded two participants with any missing covariate information. Final study population included affected probands (cases, *N* = 3350), unaffected siblings (*N* = 1759), unaffected spouses (*N* = 138), and unaffected and unrelated population-based controls (*N* = 1607). For nutrient density meat variables, estimated meat mutagen variables, and meat cooking variables from the FFQ, odds ratios (OR) and 95% confidence intervals (CI) were estimated using generalized estimating equations with robust variance estimates to predict binary outcome. This approach allowed for the nonindependence of members of the same family 37. All models were adjusted for age (years, continuous), BMI (<25, 25.0–29.9, ≥30 kg/m^2^), sex, race (NHW, non-Hispanic white; AA, African-American; Asian and others), saturated fat (g/1000 kcal/day), dietary fiber (g/1000 kcal/day), center, vegetables (g/1000 kcal/day), physical activity (h/week, continuous), and total energy intake (kcal/day, continuous). We further adjusted for potential confounding factors including income (categorical), education (categorical), smoking (current/past/never), NSAID use (current/past/never), medical history of diabetes, ulcerative colitis, Crohn's disease, irritable bowel syndrome, diverticulitis, alcohol (g/1000 kcal/day), dietary folate (g/1000 kcal/day), fruit (g/1000 kcal/day). However, results for the fully adjusted model are not shown because risk estimates did not change by >10%. Risk estimates for quintile/quartile categories of meat variables were estimated for: CRC; colon cancer (ICD-O-2 C180-C188); rectal cancer (ICD-O-2 C199, C209); MMR-proficient cancers; and MMR-deficient cancers. For all analyses, tests for trend were performed by assigning median values to each quartile/quintile category and modeling these categories as a continuous variable. Subtype-specific estimates for tumor site (colon vs. rectal cancer) or MMR status (proficient vs. deficient) were computed using all controls. Heterogeneity of risk estimates between colon versus rectal cancers and MMR-proficient versus MMR-deficient cancers was tested using case-only analyses. For analyses of effect modification by use of marinades of the association between total red meat and chicken intake, we conducted interaction tests using logistic regression and likelihood ratio tests, using energy-adjusted variables for nonprocessed red meat and nonprocessed poultry using the nutrient density method (in g/1000 kcal/day) that were categorized into quintiles, and categorical variables for the frequency of use of the two types of marinades (BBQ sauce and shoyu sauce). These interaction models were adjusted for age (years, continuous), BMI (<25, 25.0–29.9, ≥30 kg/m^2^), sex, race (NHW, non-Hispanic white; AA, African-American; Asian and others), saturated fat (g/1000 kcal/day), dietary fiber (g/1000 kcal/day), center, vegetables (g/1000 kcal/day), physical activity (h/week, continuous), and total energy intake (kcal/day, continuous). All statistical tests were two-sided and all analyses were done using Stata S/E 11.0 (STATA Corporation, College Station, TX).

## Results

Table 1 summarizes demographic, lifestyle, and key dietary characteristics of cases and controls. Cases and all types of controls were comparable in terms of age and gender, with the exception of sibling controls who were slightly younger than cases and spouse and population-based controls. There were more African-Americans (17%) among cases than among all controls combined (6%) and a higher number of non-Hispanic whites (NHW) among all controls combined (76%) than among cases (63%). Below we summarize our findings for meats main effects. In interpreting our findings we considered the fact that we tested for 27 variables that capture various combinations of meat types and cooking methods. Therefore, we applied a Bonferroni correction and regarded as our most salient findings those associations with CRC risk with trend *P*-values <0.002 (0.005/27 tests). We also note associations with *P*_trend_ <0.05 but greater than the threshold *P*-value as “suggestive” but compatible with chance. For subgroup analyses by tumor localization (colon vs. rectum) and MMR status (proficient vs. deficient), we only regarded as noteworthy those findings that showed a test of heterogeneity with *P* < 0.05, and subgroup tests for trend <0.002. All other associations with heterogeneity tests with <0.05 and test for trend <0.05 but greater than 0.002 were considered “suggestive” but compatible with chance.

**Table 1:** Demographic, lifestyle, and dietary characteristics of cases and controls in the Colon CFR

	All cases	MMR-proficient cases	MMR-deficient cases	All controls	Sibling controls	Spouse controls	Population controls
	*N* = 3350	*N* = 876	*N* = 243	*N* = 3504	*N* = 1759	*N* = 138	*N* = 1607
Characteristics	*N* (%)	*N* (%)	*N* (%)	*N* (%)	*N* (%)	*N* (%)	*N* (%)
Mean Age	59.4 ± 11.4	55.8 ± 12.4	58.9 ± 12.9	57.8 ± 11.4	55.1 ± 11.9	58.1 ± 13.5	60.6 ± 9.8
Gender							
Male	1529 (46)	483 (55)	97 (40)	1623 (46)	725 (41)	59 (42.8)	839 (52.2)
Female	1821 (54)	393 (45)	146 (60)	1881 (54)	1034 (59)	79 (57.2)	768 (47.8)
Race							
Non-Hispanic white	2096 (63)	643 (73)	208 (86)	2681 (76)	1171 (67)	28 (20.3)	1482 (92.2)
African-American	570 (17)	3 (0)	2 (1)	198 (6)	194 (11)	2 (1.4)	2 (0.1)
Asian	472 (14)	169 (19)	25 (10)	429 (12)	284 (16)	87 (63.0)	58 (3.6)
Other	212 (6)	61 (7)	8 (3)	196 (6)	110 (6)	21 (15.2)	65 (4.0)
Center							
Ontario	1855 (55)	657 (75)	166 (68)	2572 (73)	965 (54.9)	0	1607 (100.0)
Los Angeles	1059 (32)	48 (5)	54 (22)	471 (14)	471 (26.8)	0	0
Hawaii	436 (13)	171 (20)	23 (10)	461 (13)	323 (18.4)	138 (100.0)	0
BMI							
<25	1205 (36)	336 (38)	108 (44)	1471 (42)	717 (41)	70 (51)	684 (43)
25–29.9	1274 (38)	325 (37)	91 (37)	1314 (38)	625 (36)	45 (33)	644 (40)
≥30 and <55	781 (23)	189 (22)	41 (17)	657 (19)	383 (22)	21 (15)	253 (16)
Abnormal (outside 17–55)	90 (3)	26 (3)	3 (1)	62 (2)	34 (2)	2 (1)	26 (2)
Saturated fat (g/kcal per day)	11.9 ± 2.8	11.9 ± 2.8	12.3 ± 2.8	11.8 ± 2.9	11.9 ± 2.8	10.4 ± 2.5	11.9 ± 2.9
Dietary fiber (g/kcal per day)	11.2 ± 3.9	10.9 ± 3.7	11.3 ± 3.8	11.5 ± 4.0	11.2 ± 3.9	10.7 ± 4.0	11.9 ± 4.1
Total vegetables (g/kcal per day)	2.1 ± 1.0	2.0 ± 1.0	2.2 ± 1.1	2.2 ± 1.0	2.1 ± 1.1	2.1 ± 1.1	2.2 ± 1.0
Physical activity (h/month)	22.8 ± 38.9	23.9 ± 38.7	28.7 ± 50.4	24.7 ± 42.1	22.4 ± 36.9	27.4 ± 62.0	26.9 ± 45.1
Total caloric intake (kcal/day)	2321 ± 1004	2400 ± 961	2292 ± 990	2182 ± 902	2173 ± 911	2242 ± 1009	2186 ± 883
Total red meat (g/kcal per day)	30.6 ± 15.6	31.1 ± 16.3	30.0 ± 15.3	29.7 ± 15.3	30.4 ± 15.6	31.3 ± 16.1	28.8 ± 14.8
Red meat nonprocessed	20.4 ± 11.4	20.5 ± 11.6	20.6 ± 12.1	20.1 ± 11.2	20.5 ± 11.5	21.9 ± 11.9	19.5 ± 10.9
Red meat processed	10.2 ± 7.6	10.6 ± 7.8	9.4 ± 6.7	9.6 ± 7.5	10.0 ± 7.8	9.4 ± 6.2	9.3 ± 7.2
Beef	14.6 ± 8.7	14.8 ± 8.7	15.0 ± 8.8	14.5 ± 8.5	14.9 ± 8.9	15.5 ± 8.8	13.9 ± 8.0
Pork	5.0 ± 4.3	4.9 ± 4.3	4.7 ± 4.7	4.7 ± 4.2	4.8 ± 4.1	5.9 ± 4.1	4.6 ± 4.3
Sausage/lunchmeats	0.24 ± 0.21	0.25 ± 0.22	0.22 ± 0.21	0.23 ± 0.21	0.24 ± 0.22	0.20 ± 0.15	0.21 ± 0.20
Organ meat	0.02 ± 0.04	0.02 ± 0.03	0.02 ± 0.03	0.02 ± 0.04	0.01 ± 0.03	0.00 ± 0.01	0.02 ± 0.04
Total poultry (g/kcal per day)	18.2 ± 15.0	17.0 ± 12.9	15.2 ± 11.5	17.2 ± 12.8	18.5 ± 13.6	17.5 ± 12.7	15.7 ± 11.8
Poultry nonprocessed	n/a	n/a	n/a	n/a	n/a	n/a	n/a
Processed poultry	1.3 ± 2.4	1.3 ± 2.6	1.2 ± 2.8	1.1 ± 2.3	1.3 ± 2.6	0.8 ± 1.1	1.0 ± 2.1
Total HCA (g/kcal per day)	280.7 ± 325.8	267.1 ± 288.6	277.1 ± 302.9	262.8 ± 288.4	277.9 ± 306.1	209.0 ± 188.7	250.9 ± 274.1
PhIP	226.3 ± 271.1	215.8 ± 237.7	219.6 ± 250.7	212.9 ± 240.9	225.9 ± 259.2	170.2 ± 158.0	202.2 ± 224.7
MeIQx	50.9 ± 61.8	48.0 ± 57.9	53.9 ± 58.7	46.7 ± 54.8	48.6 ± 55.3	36.4 ± 35.2	45.5 ± 55.6
DiMeIQx	3.4 ± 5.2	3.3 ± 4.1	3.7 ± 4.4	3.2 ± 4.1	3.3 ± 3.9	2.3 ± 2.4	3.2 ± 4.3

Colon CFR, Colorectal Cancer Family Registry; MMR, mismatch repair; PhIP, 2-amino-1-methyl-6-phenylimidazo(4,5-b)pyridine; MeIQx, 3,8-dimethyl-3H-imidazo[4,5-f]quinoxalin-2-amine; DiMeIQx, 3,4,8-trimethyl-3H-imidazo[4,5-f]quinoxalin-2-amine; HCA, heterocyclic amines.

### Nonprocessed red meat and poultry and CRC risk

When ignoring cooking methods, we did not find statistically significant trends for any of the three specific nonprocessed red meats (beef, pork, organ meat) considered or for total nonprocessed red meat. Similarly, we did not observe statistically significant heterogeneity of effects by tumor localization or MMR status. Estimates of associations for CRC did not change for any of the abovementioned variables when restricting the analyses to those individuals likely to have sporadic CRC, given their tumor characteristics and absence of strong family history of CRC as defined the Amsterdam criteria (data not shown). We also conducted analyses restricting comparisons of cases to unrelated population-based controls, who were available from one CFR center only (Table S1). We observed similar risk estimates for all meat variables except for pork, which showed a positive association with CRC risk (fifth quintile vs. first quintile OR = 1.2; 95% CI = 1.0–1.6; *P*_trend_ = 0.028), although this trend was compatible with chance when applying a Bonferroni correction (Table S1). For all nonprocessed poultry combined, we did not see evidence of an association between diets high in poultry and CRC risk (Table 2).

**Table 2:** Meat intake and colorectal cancer risk, by cancer site and MSI status

				Cancer site							MMR status						
	Colorectal Cancer			Colon cancer			Rectal cancer				MMR-proficient			MMR-deficient			
Meat variables (g/1000 kcal/day)	CO/CA	OR^1^	95% CI	Cases	OR^1^	95% CI	Cases	OR^1^	95% CI	Het *P*	Cases	OR^1^	95% CI	Cases	OR^1^	95% CI	Het *P*
Total nonprocessed red meat																	
Q1:0–10.81	702/633	1.0^REF^		396	1.0^REF^		151	1.0^REF^			171	1.0^REF^		47	1.0^REF^		
Q2:10.81–16.04	700/644	1.0	0.9–1.2	380	1.1	0.9–1.3	168	1.0	0.8–1.3		152	0.8	0.6–1.0	47	0.9	0.6–1.4	
Q3:16.04–21.11	700/707	1.2	1.0–1.4	429	1.2	1.0–1.5	199	1.3	1.0–1.6		201	1.0	0.8–1.3	53	1.1	0.7–1.7	
Q4:21.12–28.19	701/680	1.2	1.0–1.4	396	1.2	1.0–1.4	187	1.1	0.8–1.4		179	0.8	0.7–1.1	47	1.0	0.6–1.5	
Q5:28.19–102.43	701/686	1.2	1.0–1.4	391	1.2	0.9–1.4	202	1.2	0.9–1.5		173	0.8	0.6–1.0	49	1.0	0.6–1.7	
*P*_trend_			0.085			0.152			0.315	0.781			0.104			0.835	0.265
Beef																	
Q1:0–7.69	702/687	1.0^REF^		436	1.0^REF^		155	1.0^REF^			167	1.0^REF^		42	1.0^REF^		
Q2:7.7–11.49	699/652	1.0	0.9–1.2	377	0.9	0.8–1.1	185	1.2	0.9–1.5		165	0.9	0.7–1.1	54	1.2	0.8–1.9	
Q3:11.49–15.08	701/654	1.0	0.9–1.2	396	1.0	0.8–1.2	174	1.1	0.8–1.4		171	0.9	0.7–1.1	46	1.0	0.7–1.6	
Q4:15.09–20.06	701/672	1.1	0.9–1.3	400	1.1	0.9–1.3	184	1.1	0.9–1.4		192	0.9	0.7–1.2	55	1.3	0.8–2.0	
Q5:20.08–83.77	701/685	1.1	0.9–1.3	383	1.0	0.8–1.2	209	1.2	0.9–1.6		181	0.9	0.7–1.1	46	1.0	0.7–1.7	
*P*_trend_			0.289			0.593			0.252	0.292			0.414			0.968	0.759
Pork																	
Q1:0–1.32	702/617	1.0^REF^		383	1.0^REF^		154	1.0^REF^			159	1.0^REF^		50	1.0^REF^		
Q2:1.32–3.01	700/641	1.0	0.9–1.2	388	1.0	0.8–1.2	163	1.0	0.8–1.3		180	1.0	0.8–1.3	50	1.0	0.6–1.4	
Q3:3.01–4.84	701/660	1.1	0.9–1.2	383	1.0	0.9–1.2	178	1.1	0.8–1.3		160	0.9	0.7–1.1	49	1.0	0.6–1.5	
Q4:4.85–7.44	701/743	1.2	1.0–1.4	440	1.2	1.0–1.4	207	1.2	0.9–1.5		195	1.0	0.8–1.3	53	1.0	0.7–1.6	
Q5:7.44–49.62	700/689	1.1	1.0–1.3	398	1.1	0.9–1.3	205	1.1	0.9–1.5		182	0.9	0.7–1.2	41	0.9	0.6–1.4	
*P*_trend_			0.069			0.224			0.133	0.562			0.585			0.714	0.837
Organ meat																	
Q1:0–0	1124/884	1.0^REF^		554	1.0^REF^		206	1.0^REF^			250	1.0^REF^		79	1.0^REF^		
Q2:0–0	278/282	1.2	1.0–1.4	152	1.1	0.8–1.4	91	1.4	1.0–1.9		91	1.1	0.8–1.5	20	0.9	0.5–1.5	
Q3:0–0	700/650	1.1	1.0–1.3	382	1.1	0.9–1.3	176	1.3	1.0–1.6		178	1.0	0.8–1.3	49	1.0	0.7–1.5	
Q4:0–0.02	701/755	1.0	0.9–1.2	431	0.9	0.8–1.1	233	1.4	1.1–1.7		181	0.9	0.7–1.2	38	0.8	0.5–1.2	
Q5:0.02–0.64	701/779	1.2	1.0–1.4	473	1.2	1.0–1.4	201	1.3	1.0–1.6		176	1.0	0.8–1.3	57	1.1	0.7–1.6	
*P*_trend_			0.058			0.02			0.47	0.215			0.708			0.346	0.205
Processed red meat																	
Q1:0–3.97	701/592	1.0^REF^		347	1.0^REF^		152	1.0^REF^			151	1.0^REF^		46	1.0^REF^		
Q2:3.97–6.74	701/659	1.1	0.9–1.2	411	1.2	1.0–1.4	155	0.9	0.7–1.2		154	0.9	0.7–1.2	64	1.3	0.9–2.0	
Q3:6.75–9.53	701/618	1.0	0.8–1.2	368	1.0	0.9–1.3	165	0.9	0.7–1.2		157	0.9	0.7–1.1	41	0.9	0.5–1.3	
Q4:9.53–13.86	701/688	1.1	0.9–1.3	396	1.1	0.9–1.3	220	1.2	0.9–1.5		202	1.1	0.8–1.4	39	0.8	0.5–1.3	
Q5:13.87–122.42	700/793	1.1	1.0–1.4	470	1.2	1.0–1.5	215	1.0	0.8–1.4		212	1.1	0.8–1.4	53	1.1	0.7–1.8	
*P*_trend_			0.105			0.092			0.277	0.733			0.136			0.929	0.34
Sausage and lunchmeats																	
Q1:0–0.08	702/582	1.0^REF^		350	1.0^REF^		142	1.0^REF^			138	1.0^REF^		44	1.0^REF^		
Q2:0.08–0.14	700/657	1.1	0.9–1.3	395	1.1	0.9–1.4	169	1.1	0.8–1.4		148	1.0	0.7–1.3	58	1.3	0.8–1.9	
Q3:0.14–0.22	701/706	1.2	1.0–1.4	409	1.2	1.0–1.4	190	1.1	0.9–1.5		194	1.2	1.0–1.6	56	1.2	0.8–1.9	
Q4:0.22–0.32	700/654	1.0	0.9–1.2	396	1.1	0.9–1.3	195	1.1	0.9–1.5		179	1.1	0.8–1.4	40	0.9	0.6–1.4	
Q5:0.32–3.86	701/751	1.2	1.0–1.4	442	1.2	1.0–1.4	211	1.2	0.9–1.5		217	1.3	1.0–1.7	45	1.0	0.6–1.6	
*P*_trend_			0.187			0.177			0.231	0.86			0.029			0.408	0.069
Poultry																	
Q1:0–7.86	702/634	1.0^REF^		372	1.0^REF^		183	1.0^REF^			173	1.0^REF^		55	1.0^REF^		
Q2:7.86–11.86	700/642	1.0	0.9–1.2	384	1.1	0.9–1.3	166	0.9	0.7–1.1		192	1.1	0.9–1.4	63	1.2	0.8–1.7	
Q3:11.86–16.73	701/687	1.0	0.9–1.2	393	1.0	0.9–1.2	189	1.0	0.8–1.2		183	1.0	0.8–1.3	49	0.9	0.6–1.4	
Q4:16.73–24.66	701/664	1.0	0.8–1.2	396	1.0	0.8–1.2	180	0.9	0.7–1.2		165	0.9	0.7–1.2	43	0.8	0.5–1.2	
Q5:24.67–230.97	700/723	0.9	0.8–1.1	447	1.0	0.8–1.2	189	0.9	0.7–1.1		163	0.9	0.7–1.2	33	0.6	0.4–1.0	
*P*_trend_			0.362			0.46			0.485	0.807			0.368			0.017	0.151
Processed poultry																	
Q1:0–0.04	702/590	1.0^REF^		353	1.0^REF^		160	1.0^REF^			142	1.0^REF^		43	1.0^REF^		
Q2:0.04–0.15	701/646	1.1	0.9–1.2	362	1	0.9–1.2	188	1.1	0.8–1.4		161	1.0	0.8–1.3	60	1.4	0.9–2.1	
Q3:0.15–0.63	701/698	1.1	0.9–1.3	407	1.1	0.9–1.3	186	1.1	0.8–1.3		184	1.2	0.9–1.5	48	1.1	0.7–1.7	
Q4:0.63–1.65	699/657	1.0	0.8–1.1	395	1.0	0.8–1.2	166	0.9	0.7–1.1		182	1.1	0.9–1.4	45	1.0	0.7–1.6	
Q5:1.65–37.93	701/759	1.1	1.0–1.3	475	1.2	1.0–1.4	207	1.1	0.9–1.4		207	1.3	1.0–1.6	47	1.0	0.7–1.6	
*P*_trend_			0.339			0.055			0.794	0.214			0.046			0.548	0.166
Total processed meats (red meat + poultry)																	
Q1:0–4.43	702/593	1.0^REF^		346	1.0^REF^		155	1.0^REF^			146	1.0^REF^		44	1.0^REF^		
Q2:4.43–7.35	700/643	1.1	0.9–1.2	395	1.2	1.0–1.4	159	0.9	0.7–1.2		154	1.0	0.8–1.3	58	1.3	0.8–1.9	
Q3:7.36–10.62	701/640	1.0	0.9–1.2	383	1.1	0.9–1.3	165	0.9	0.7–1.1		161	0.9	0.7–1.2	53	1.1	0.7–1.8	
Q4:10.63–15.29	701/654	1.0	0.8–1.2	385	1.1	0.9–1.3	200	1.0	0.8–1.3		197	1.1	0.9–1.4	36	0.8	0.5–1.3	
Q5:15.29–152.04	700/820	1.2	1.0–1.4	483	1.3	1.0–1.5	228	1.1	0.8–1.4		218	1.2	0.9–1.5	52	1.2	0.7–1.8	
*P*_trend_			0.054			0.05			0.2	0.715			0.052			0.942	0.249

CO, controls; CA, cases; MMR, mismatch repair; MSI, microsatellite instability; Het *P*:*P*-value of test of heterogeneity.

1 OR adjusted for age (years, continuous), BMI (<25, 25.0–29.9, ≥30), gender, race (NHW, AA, Asian and others), saturated fat (g/1000 kcal/day), dietary fiber (g/1000 kcal/day), center, vegetables (g/1000 kcal/day), physical activity (h/week, continuous), and total calorie intake (kcal/day, continuous).

We next considered the modifying effect of frequency of intake of two different types of marinades (BBQ sauce and shoyu) on the association between nonprocessed red meat and nonprocessed poultry with CRC risk. We observed evidence of effect modification of the association between nonprocessed red meat and CRC risk by frequency of use of shoyu marinade. Specifically, we observed that a positive association between increasing levels of nonprocessed red meat and CRC risk was restricted to individuals who reported never eating shoyu marinade (fifth quintile vs. first quintile OR = 1.3; 95% CI = 1.1–1.6; *P*_trend_ = 0.007). In contrast, no associations were observed among individuals with low (once a month or less) or high (more than once a month) use of shoyu (test of heterogeneity of trends *P* = 0.008) (Table S2). We also observed some evidence that the frequency of intake of BBQ sauce modified the association between high nonprocessed red meat intake and CRC risk. Specifically, we observed that this association was slightly stronger among individuals who reported using BBQ sauce marinade more than once a month (fifth quintile vs. first quintile OR = 1.5; 95% CI = 0.9–2.4; *P*_trend_ = 0.01). Although positive associations were observed between some levels of intake of nonprocessed red meat and CRC risk among individuals who reported never using BBQ sauce, the trend was not statistically significant. No comparable trend was observed among individuals who reported using BBQ sauce less than once per month. However, the test of heterogeneity of trends did not reach statistical significance (*P* = 0.084) (Table S2). No differences in trends were observed for nonprocessed poultry for use of BBQ sauce or shoyu marinade.

### Processed red meat and poultry and CRC risk

For total processed red meat, no statistically significant associations with CRC were observed, and there was no statistically significant heterogeneity between colon and rectal tumors, or by MMR status. There was no statistically significant trend between diets high in sausages and lunchmeats and CRC risk; however, we observed for these foods a statistically significant positive association among MMR-proficient CRC tumors (fifth quintile vs. first quintile OR = 1.3; 95% CI = 1.0–1.7; *P*_trend_ = 0.029), which was compatible with chance when considering a Bonferroni correction, but the test for heterogeneity was not statistically significant (*P* = 0.069) (Table 2). Similarly, there was no association between diets high in processed poultry and CRC risk, but we observed a significant positive association for these foods with among MMR-proficient CRC tumors (fifth quintile vs. first quintile OR = 1.3; 95% CI = 1.0–1.6; *P*_trend_ = 0.046), which was also compatible with chance when considering a Bonferroni correction, and no statistically significant test of heterogeneity (*P* = 0.166) (Table 2).

In general, similar trends and estimates were observed when restricting analyses to cases and population-based controls (Table S1). Moreover, estimates of associations for CRC did not change for either processed meat variable when restricting analyses to those individuals without familial history of CRC, as defined by the Amsterdam criteria (data not shown).

### Pan-fried meats and CRC risk

We investigated the association between CRC risk and frequency of intake of any of the following meats cooked by pan-frying (defined as “cooked in a preheated frying pan or griddle”): beefsteak, hamburger, chicken, sausage, spam or ham, or bacon. We observed a statistically significant positive association between intake of beefsteak and CRC risk (*P*_trend_ < 0.0002), which was not compatible with chance after Bonferroni correction (Table 3). Even though this association seemed stronger for colon (*P*_trend_ = 0.0005) than rectal cancer (*P*_trend_ = 0.09), the test of heterogeneity was not statistically significant. Moreover, this association with beefsteak seemed stronger among MMR-deficient cases (*P*_trend_ = 0.002; heterogeneity *P* = 0.059). We also noted positive associations between intake of pan-fried sausage and spam or ham and CRC risk, which were statistically significant, albeit compatible with chance when considering a Bonferroni correction (*P*_trend_ = 0.04 and *P*_trend_ = 0.048, respectively). However, the association with spam or ham was stronger when analyses were restricted to MMR-proficient cases, and not compatible with chance when considering a Bonferroni correction (*P*_trend_ = 0.0001; heterogeneity *P* = 0.026). Further consideration of level of doneness for each of these variables did not show appreciable differences between light- or medium-brown versus dark-brown meats. Restricting analyses to cases and population-based controls showed similar trends and estimates (Table S1). Similarly, restricting analyses to cases without family history of CRC (Amsterdam criteria), resulted in similar estimates (data not shown).

**Table 3:** Pan-fried meat intake and colorectal cancer risk, by cancer site and MSI status

				Cancer site							MMR status							
	Colorectal cancer			Colon cancer			Rectal cancer				MMR-proficient			MMR-deficient			
Meat variables (servings/1000 kcal/day)	CO/CA	OR^1^	95% CI	Cases	OR^1^	95% CI	Cases	OR^1^	95% CI	Het *P*	Cases	OR^1^	95% CI	Cases	OR^1^	95% CI	Het *P*
Beef steak																	
Q1:0–0	1967/1692	1.0^REF^		1019	1.0^REF^		449	1.0^REF^			469	1.0^REF^		121	1.0^REF^		
Q2:0.01–0.02	496/506	1.0	0.8–1.1	300	1.0	0.8–1.1	146	1.0	0.8–1.2		129	0.9	0.7–1.1	33	1.0	0.7–1.5	
Q3:0.02–0.04	497/511	1.0	0.9–1.2	287	0.9	0.8–1.1	152	1.1	0.9–1.4		119	0.9	0.7–1.1	35	1.1	0.8–1.7	
Q4:0.04–0.99	496/619	1.3	1.1–1.5	374	1.4	1.2–1.6	152	1.2	1.0–1.5		155	1.2	1.0–1.5	54	1.7	1.2–2.4	
*P*_trend_			<0.001			<0.001			0.09	0.379			0.098			0.002	0.059
Hamburger																	
Q1:0–0	1511/1297	1.0^REF^		778	1.0^REF^		352	1.0^REF^			381	1.0^REF^		89	1.0^REF^		
Q2:0.01–0.02	655/627	0.9	0.8–1.1	369	0.9	0.8–1.1	169	0.9	0.7–1.1		164	0.8	0.7–1.0	34	0.8	0.5–1.2	
Q3:0.02–0.05	654/707	1.0	0.9–1.2	420	1.1	0.9–1.3	194	1.0	0.8–1.3		178	1.0	0.8–1.2	56	1.3	0.9–1.9	
Q4:0.05–0.99	655/697	1.1	0.9–1.2	410	1.1	0.9–1.2	186	1.1	0.9–1.4		150	0.9	0.7–1.1	63	1.5	1.0–2.1	
*P*_trend_			0.209			0.354			0.198	0.516			0.516			0.01	0.026
Chicken																	
Q1:0–0	1374/1096	1.0^REF^		656	1.0^REF^		302	1.0^REF^			312	1.0^REF^		98	1.0^REF^		
Q2:0.01–0.03	699/653	0.9	0.8–1.1	386	0.9	0.8–1.1	184	0.9	0.7–1.1		194	1.0	0.8–1.3	40	0.7	0.5–1.1	
Q3:0.03–0.07	700/781	1.2	1.0–1.3	453	1.1	1.0–1.3	215	1.2	0.9–1.4		199	1.1	0.9–1.4	53	1.1	0.8–1.6	
Q4:0.07–1.18	698/788	1.1	0.9–1.2	475	1.1	0.9–1.2	197	1.1	0.9–1.3		163	1.0	0.8–1.2	52	1.1	0.8–1.6	
*P*_trend_			0.13			0.352			0.322	0.94			0.856			0.401	0.164
Sausage																	
Q1:0–0	1530/1271	1.0^REF^		789	1.0^REF^		302	1.0^REF^			330	1.0^REF^		101	1.0^REF^		
Q2:0.01–0.02	652/643	1.1	1.0–1.3	371	1.1	0.9–1.3	204	1.3	1.1–1.6		205	1.2	1.0–1.5	46	1.0	0.7–1.5	
Q3:0.02–0.04	650/619	1.1	0.9–1.2	356	1.0	0.8–1.2	177	1.2	1.0–1.5		161	1.0	0.8–1.2	46	1.0	0.7–1.5	
Q4:0.04–1.32	650/781	1.2	1.0–1.3	456	1.1	0.9–1.3	213	1.4	1.1–1.7		172	1.2	0.9–1.4	50	1.2	0.8–1.7	
*P*_trend_			0.041			0.371			0.004	0.053			0.318			0.325	0.527
Spam or ham																	
Q1:0–0	2414/2097	1.0^REF^		1240	1.0^REF^		556	1.0^REF^			524	1.0^REF^		173	1.0^REF^		
Q2:0.01–0.02	345/395	1.0	0.9–1.2	232	1.0	0.9–1.3	121	1.1	0.9–1.5		106	1.3	1.0–1.7	18	0.6	0.4–1.0	
Q3:0.02–0.04	347/403	1.1	0.9–1.3	245	1.1	0.9–1.3	109	1.1	0.9–1.4		110	1.4	1.1–1.8	30	1.1	0.7–1.6	
Q4:0.04–0.99	345/425	1.2	1.0–1.4	256	1.2	1.0–1.4	117	1.3	1.0–1.6		128	1.6	1.2–2.0	19	0.8	0.5–1.3	
*P*_trend_			0.048			0.107			0.064	0.965			<0.001			0.461	0.026
Bacon																	
Q1:0–0	1237/1094	1.0^REF^		676	1.0^REF^		268	1.0^REF^			292	1.0^REF^		80	1.0^REF^		
Q2:0.01–0.03	750/664	1.0	0.8–1.1	374	0.9	0.8–1.1	212	1.1	0.9–1.4		202	0.9	0.8–1.2	56	1.0	0.7–1.5	
Q3:0.03–0.05	748/720	1.0	0.9–1.2	429	1.0	0.9–1.2	206	1.1	0.9–1.4		216	1.1	0.9–1.4	54	0.9	0.6–1.4	
Q4:0.05–1.43	748/841	1.0	0.9–1.2	497	1.0	0.8–1.2	209	1.1	0.9–1.3		160	0.9	0.7–1.1	53	1.0	0.7–1.4	
*P*_trend_			0.61			0.762			0.76	0.921			0.478			0.744	0.937
Total pan-fried meat intake																	
Q1:0–0.05	842/638	1.0^REF^		380	1.0^REF^		168	1.0^REF^			195	1.0^REF^		45	1.0^REF^		
Q2:0.05–0.12	841/739	1.1	0.9–1.3	434	1.1	0.9–1.3	216	1.1	0.9–1.4		219	1.0	0.8–1.3	62	1.3	0.9–2.0	
Q3:0.12–0.21	842/820	1.1	0.9–1.3	499	1.1	1.0–1.3	225	1.1	0.9–1.4		224	1.0	0.8–1.3	61	1.3	0.9–2.0	
Q4:0.21–5.96	842/1051	1.2	1.1–1.4	622	1.2	1.0–1.4	266	1.3	1.0–1.6		212	1.1	0.8–1.3	71	1.5	1.0–2.3	
*P*_trend_			0.007			0.039			0.059	0.904			0.6			0.054	0.072

CO, controls; CA, cases; MMR, mismatch repair; MSI, microsatellite instability; Het *P*:*P*-value of test of heterogeneity.

1 OR adjusted for age (years, continuous), BMI (<25, 25.0–29.9, ≥30), gender, race (NHW, AA, Asian and others), saturated fat (g/1000 kcal/day), dietary fiber (g/1000 kcal/day), center, vegetables (g/1000 kcal/day), physical activity (h/week, continuous), and total calorie intake (kcal/day, continuous).

### Oven-broiled and grilled meats and CRC risk

We investigated the association between CRC risk and any of the following meats cooked by oven-broiling, defined as “cooked at the broil setting”: beefsteak, hamburgers, chicken, and short ribs or spareribs. We observed a positive association between frequent intake of oven-broiled short ribs or spareribs and CRC risk (fourth quartile vs. first quartile OR = 1.2; 95% CI = 1.0–1.5; *P*_trend_ = 0.0002), which remained of borderline statistical significance after Bonferroni correction, without evidence of heterogeneity by tumor site (Table 4). This association was restricted to MMR-deficient cases (fourth quartile vs. first quartile OR = 1.9; 95% CI = 1.2–3.0; *P*_trend_ = 0.003; tests of heterogeneity *P* = 0.05) (Table 4). Similar trends and estimates were observed when restricting analyses to cases and population-based controls (Table S1).

**Table 4:** Oven-broiled meat intake and colorectal cancer risk, by cancer site and MSI status

				Cancer site							MMR status						
	Colorectal cancer			Colon cancer			Rectal cancer				MMR-proficient			MMR-deficient			
Meat variables (servings/1000 kcal/day)	CO/CA	OR^1^	95% CI	Cases	OR^1^	95% CI	Cases	OR^1^	95% CI	Het *P*	Cases	OR^1^	95% CI	Cases	OR^1^	95% CI	Het *P*
Beefsteak																	
Q1:0–0	2363/2145	1.0^REF^		1297	1.0^REF^		555	1.0^REF^			569	1.0^REF^		159	1.0^REF^		
Q2:0.01–0.02	356/399	1.0	0.8–1.2	226	1.0	0.8–1.2	128	1.2	0.9–1.5		111	1.1	0.8–1.4	23	0.9	0.5–1.4	
Q3:0.02–0.04	358/397	1.1	0.9–1.3	233	1.0	0.9–1.2	110	1.2	0.9–1.5		104	1.2	0.9–1.5	22	0.9	0.6–1.4	
Q4:0.04–1.37	356/346	0.9	0.8–1.1	203	0.9	0.8–1.1	93	1.1	0.8–1.4		76	1.0	0.7–1.3	36	1.5	1.0–2.1	
*P*_trend_			0.742			0.507			0.333	0.228			0.82			0.122	0.221
Hamburger																	
Q1:0–0	2718/2506	1.0^REF^		1478	1.0^REF^		679	1.0^REF^			688	1.0^REF^		184	1.0^REF^		
Q2:0.01–0.02	247/241	0.8	0.7–1.0	134	0.8	0.6–1.0	75	0.9	0.7–1.2		60	0.8	0.6–1.1	8	0.4	0.2–0.9	
Q3:0.02–0.04	248/279	1.0	0.8–1.2	181	1.1	0.9–1.3	75	1.1	0.8–1.4		55	0.9	0.7–1.2	19	1.0	0.6–1.7	
Q4:0.04–0.99	249/283	1.0	0.9–1.2	178	1.1	0.9–1.3	65	1.0	0.7–1.3		61	1.1	0.8–1.6	28	1.6	1.0–2.4	
*P*_trend_			0.989			0.535			0.89	0.71			0.731			0.094	0.342
Chicken																	
Q1:0–0	1788/1585	1.0^REF^		932	1.0^REF^		425	1.0^REF^			408	1.0^REF^		127	1.0^REF^		
Q2:0.01–0.03	565/568	1.0	0.9–1.2	335	1.0	0.9–1.2	175	1.0	0.8–1.3		163	1.0	0.8–1.3	26	0.6	0.4–1.0	
Q3:0.03–0.06	565/581	1.1	1.0–1.3	348	1.1	0.9–1.3	151	1.1	0.9–1.3		164	1.2	1.0–1.5	42	1.1	0.8–1.6	
Q4:0.06–1.33	564/585	1.1	0.9–1.2	365	1.1	0.9–1.3	146	1.1	0.9–1.3		133	1.1	0.9–1.4	46	1.3	0.9–1.8	
*P*_trend_			0.291			0.169			0.537	0.675			0.207			0.128	0.477
Short ribs or spareribs																	
Q1:0–0	2747/2389	1.0^REF^		1423	1.0^REF^		627	1.0^REF^			656	1.0^REF^		178	1.0^REF^		
Q2:0.01–0.02	236/319	1.2	1.0–1.5	175	1.2	0.9–1.5	115	1.6	1.2–2.0		91	1.3	1.0–1.7	15	0.9	0.5–1.5	
Q3:0.02–0.03	239/299	1.3	1.1–1.6	189	1.4	1.1–1.7	74	1.2	0.9–1.6		64	1.1	0.8–1.5	21	1.5	0.9–2.4	
Q4:0.03–0.99	237/306	1.2	1.0–1.5	189	1.2	1.0–1.5	78	1.3	1.0–1.7		58	1.0	0.8–1.4	26	1.9	1.2–3.0	
*P*_trend_			0.002			0.002			0.007	0.817			0.415			0.003	0.052
Total oven-broiled meat intake																	
Q1:0–0	1446/1237	1.0^REF^		738	1.0^REF^		320	1.0^REF^			335	1.0^REF^		105	1.0^REF^		
Q2:0.01–0.05	647/664	1.1	1.0–1.3	376	1.1	0.9–1.3	202	1.2	1.0–1.4		192	1.1	0.9–1.3	36	0.8	0.5–1.2	
Q3:0.05–0.1	648/618	1.0	0.9–1.2	376	1.0	0.9–1.2	163	1.0	0.8–1.2		161	1.0	0.8–1.2	38	0.8	0.5–1.2	
Q4:0.1–3.97	648/730	1.1	1.0–1.3	451	1.1	1.0–1.3	189	1.2	1.0–1.5		162	1.1	0.9–1.4	57	1.3	0.9–1.8	
*P*_trend_			0.193			0.125			0.115	0.797			0.296			0.137	0.276

CO, controls; CA, cases; MMR, mismatch repair; MSI, microsatellite instability; Het *P*:*P*-value of test of heterogeneity.

OR adjusted for age (years, continuous), BMI (<25, 25.0–29.9, ≥30), gender, race (NHW, AA, Asian and others), saturated fat (g/1000 kcal/day), dietary fiber (g/1000 kcal/day), center, vegetables (g/1000 kcal/day), physical activity (h/week, continuous), and total calorie intake (kcal/day, continuous).

We also investigated the association between CRC risk and any of the following meats cooked by grilling or barbecuing, defined as “cooked over charcoal or on an electric or gas grill”: beefsteak, hamburgers, chicken, sausage, and short ribs or spareribs. We did not observe any positive associations with CRC risk for any of these variables (Table 5). However, we observed a statistically significant inverse association between higher frequency of intake of grilled or BBQ hamburgers and CRC risk (fourth quartile vs. first quartile OR = 0.8; 95% CI = 0.7–0.9; *P*_trend_ = 0.002) (Table 5). This association was of borderline statistical significance after Bonferroni correction. Similar estimates were observed when restricting analyses to cases and population-based controls (Table S1).

**Table 5:** Grilled meat intake and colorectal cancer risk, by cancer site and MSI status

				Cancer site							MMR status						
	colorectal cancer			Colon cancer			Rectal cancer				MMR-proficient			MMR-deficient			
Meat variables (servings/1000 kcal/day)	CO/CA	OR^1^	95% CI	Cases	OR^1^	95% CI	Cases	OR^1^	95% CI	Het *P*	Cases	OR^1^	95% CI	Cases	OR^1^	95% CI	Het *P*
Beefsteak																	
Q1:0–0	1222/1314	1.0^REF^		814	1.0^REF^		295	1.0^REF^			281	1.0^REF^		78	1.0^REF^		
Q2:0.01–0.02	740/726	0.9	0.8–1.1	413	0.9	0.8–1.0	243	1.2	1.0–1.5		200	0.9	0.8–1.2	53	1.1	0.8–1.6	
Q3:0.02–0.04	744/677	1.0	0.8–1.1	401	1	0.8–1.1	187	1.0	0.8–1.2		201	1.0	0.8–1.2	56	1.2	0.8–1.7	
Q4:0.04–0.99	742/582	0.9	0.8–1.0	335	0.9	0.7–1.0	166	1.0	0.8–1.2		177	1.0	0.8–1.2	55	1.2	0.8–1.7	
*P*_trend_			0.212			0.182			0.686	0.478			0.819			0.362	0.351
Hamburger																	
Q1:0–0	1266/1401	1.0^REF^		844	1.0^REF^		332	1.0^REF^			336	1.0^REF^		81	1.0^REF^		
Q2:0.01–0.02	736/690	0.9	0.7–1.0	399	0.8	0.7–1.0	219	1.0	0.8–1.2		167	0.7	0.6–0.9	47	0.9	0.6–1.3	
Q3:0.02–0.05	736/686	0.9	0.8–1.1	407	0.9	0.8–1.1	198	1.0	0.8–1.2		207	0.9	0.8–1.2	61	1.2	0.8–1.7	
Q4:0.05–0.99	736/542	0.8	0.7–0.9	326	0.8	0.7–1.0	149	0.9	0.7–1.1		157	0.8	0.6–1.0	53	1.0	0.7–1.5	
*P*_trend_			0.002			0.038			0.173	0.81			0.196			0.586	0.272
Chicken																	
Q1:0–0	1163/1195	1.0^REF^		729	1.0^REF^		280	1.0^REF^			251	1.0^REF^		84	1.0^REF^		
Q2:0.01–0.03	770/835	1.0	0.9–1.1	482	1.0	0.8–1.1	254	1.1	0.9–1.4		232	1.2	1.0–1.5	58	1.0	0.7–1.5	
Q3:0.03–0.06	773/691	0.9	0.8–1.1	408	0.9	0.8–1.1	200	1.1	0.9–1.3		200	1.0	0.8–1.3	58	1.1	0.8–1.6	
Q4:0.06–1.15	771/604	0.9	0.8–1.0	360	0.9	0.8–1.0	168	1.0	0.8–1.2		188	1.1	0.9–1.4	43	0.8	0.6–1.2	
*P*_trend_			0.097			0.139			0.778	0.347			0.444			0.322	0.242
Sausage																	
Q1:0–0	2255/2222	1.0^REF^		1321	1.0^REF^		571	1.0^REF^			507	1.0^REF^		162	1.0^REF^		
Q2:0.01–0.02	402/410	1.1	0.9–1.3	237	1.1	0.9–1.3	134	1.1	0.9–1.4		147	1.3	1.0–1.6	28	1.0	0.6–1.5	
Q3:0.02–0.03	405/327	0.9	0.8–1.1	208	1.0	0.9–1.2	88	0.9	0.7–1.1		111	1.1	0.8–1.3	21	0.8	0.5–1.3	
Q4:0.03–0.99	404/357	1.0	0.9–1.2	209	1.0	0.8–1.2	105	1.1	0.8–1.4		104	1.1	0.8–1.4	32	1.2	0.8–1.9	
*P*_trend_			0.903			0.779			0.785	0.93			0.429			0.521	0.695
Short ribs or spareribs																	
Q1:0–0	2493/2239	1.0^REF^		1341	1.0^REF^		575	1.0^REF^			602	1.0^REF^		173	1.0^REF^		
Q2:0.01–0.02	320/360	0.9	0.8–1.1	205	0.9	0.8–1.1	117	1.0	0.8–1.3		101	1.0	0.8–1.3	19	0.7	0.4–1.2	
Q3:0.02–0.03	321/344	1.1	0.9–1.3	203	1.1	0.9–1.3	111	1.4	1.1–1.7		79	1.0	0.7–1.2	23	1.1	0.7–1.8	
Q4:0.03–0.99	322/361	1.1	0.9–1.3	221	1.2	1.0–1.4	88	1.1	0.8–1.4		83	1.0	0.8–1.3	26	1.2	0.8–1.9	
*P*_trend_			0.166			0.17			0.146	0.92			0.987			0.431	0.42
Total grilled meat intake																	
Q1:0–0	661/736	1.0^REF^		456	1.0^REF^		159	1.0^REF^			157	1.0^REF^		46	1.0^REF^		
Q2:0.01–0.07	906/944	0.9	0.8–1.1	551	0.9	0.8–1.1	273	1.1	0.8–1.3		245	0.9	0.8–1.2	65	1.0	0.7–1.5	
Q3:0.07–0.15	910/805	0.9	0.8–1.0	463	0.8	0.7–1.0	238	1.0	0.8–1.3		220	0.9	0.7–1.1	68	1.1	0.7–1.6	
Q4:0.15–4.97	907/757	0.9	0.8–1.0	465	0.9	0.8–1.1	202	1.0	0.8–1.2		224	1.0	0.8–1.2	60	1.0	0.6–1.4	
*P*_trend_			0.136			0.44			0.454	0.99			0.833			0.777	0.86

CO, controls; CA, cases; MMR, mismatch repair; MSI, microsatellite instability; Het *P*:*P*-value of test of heterogeneity.

OR adjusted for age (years, continuous), BMI (<25, 25.0–29.9, ≥30), gender, race (NHW, AA, Asian and others), saturated fat (g/1000 kcal/day), dietary fiber (g/1000 kcal/day), center, vegetables (g/1000 kcal/day), physical activity (h/week, continuous), and total calorie intake (kcal/day, continuous).

### HCA and CRC risk

We considered possible associations between CRC risk and imputed estimates of dietary intake for three different HCAs known to occur in meats: PhiP, MeIQx, and DiMeIQX. We only observed a statistically significant positive trend of association between increasing levels of DiMeIQx and MMR-deficient CRC (fifth quintile vs. first quintile OR = 1.2; 95% CI = 0.8–1.8; *P*_trend_ = 0.042; *P* for heterogeneity by MMR status = 0.17) (Table S3). However, this association was compatible with chance after Bonferroni correction. Similar trends were observed for PhIP and MeIQx, but neither were statistically significant. Slightly stronger evidence of a positive association for DiMeIQx and MMR-deficient CRC was observed when restricting analyses to cases and population-based controls (Table S1).

## Discussion

Given the comprehensive nature of our study, many tests were done. After adjusting *P*-values for multiple testing using a Bonferroni correction, our most salient finding is a positive association between CRC risk and a high intake of pan-fried beefsteak, which seemed more important for MMR-deficient tumors. We observed similar positive association with other pan-fried meats (sausage, spam, or ham), albeit none remained significant after multiple testing correction. However, we observed a positive association that was not compatible with chance for pan-fried spam or ham among MMR-proficient cases. Another salient finding was the observation of a positive association between CRC risk and frequent intake of oven-broiled short ribs, which was restricted to MMR-deficient tumors, and an inverse association between frequent intake of grilled or BBQ hamburgers and CRC risk. Both associations were of borderline statistical significance after multiple testing corrections. We found no strong associations for total nonprocessed red meat or total processed meats. Our findings suggest that consideration of individual types of meats and cooking practices is important in order to understand the meat and cancer risk association, and this may explain why other studies failed to find positive associations with total red meat 24,26,38–40 or found weak associations 27,41,42.

Pan-frying involves frying foods on a flat pan using just enough cooking oil or fat to lubricate the pan. This layer of oil acts as an efficient heat transfer medium between the pan and the surface of the meat allowing the meat to reach very high surface temperatures. Pan-frying is consistently implicated in the formation of HCAs, but not PAHs 11,43,44. Consistent with a role for HCAs in CRC risk were our findings from analyses that took into account marinades. A positive association between high intake of nonprocessed red meat and CRC risk was restricted to individuals who either never used Asian-type marinades—which have been shown to reduce HCA formation 45,46—or frequently used BBQ sauce marinade—which has been reported to increase HCA formation 45. Alternative explanations for our findings are effects due to absorption of culinary fat, free radical formation from fats, and exposure to cookware-related substances, such as perfluorooctanoate 47,48. We cannot discard other aspects of diet and lifestyle, not considered in our analyses, which may confound this association. Only four other epidemiologic studies have considered pan-fried meats separately from other cooking methods; two case–control studies reported positive associations 23,27, and one prospective 24 and another case–control study 30 reported no association. One additional case–control study investigated pan-fried meats combined with fried meats, and they reported a positive association that was statistically significant for rectal cancer 32. Previously, we reported a similar stronger association between pan-fried red meat and fish and prostate cancer, with no associations for meats cooked by other methods 49,50.

We observed a positive association with oven-broiled short ribs and CRC. In the United States, oven-baked short ribs are often cooked with BBQ sauce or comparable marinades, which have been shown to increase the formation of HCAs when meats are exposed to high temperatures 45. Also, we observed a borderline statistically significant inverse association between grilled hamburgers and CRC risk. Both findings should be explored further.

For the two meat types that we found positive associations that were not compatible with chance after multiple testing correction (pan-fried beefsteak and oven-broiled short ribs or spareribs), we observed that they were associated more strongly with MMR-deficient cancer. If the mechanism underlying this association relates to HCA exposure, our data suggest that variation in the repair of damage by the MMR pathway might be relevant for exposure to this carcinogen, which is biologically plausible given that HCAs are known to induce frameshift mutations, MSI, and base mismatches 51, which would elicit the MMR pathway. Consistent with this possibility, we observed that estimated levels of two (MeIQx and DiMeIQx) of the three investigated HCAs were positively associated with MMR-deficient CRC. However, both findings were compatible with chance when considering a multiple testing correction. Among previous epidemiologic studies that considered estimated levels of HCAs and CRC risk, two prospective studies 24,38 and one case–control study 52 reported no associations. In contrast, one prospective study reported an association between DiMeIQx and colon cancer 25, and, five case–control studies reported associations between DiMeIQx only 23 or MeIQx only 22,28, DiMeIQx and MeIQx 27, or PhIP, MeIQx and diMeIQx 31. Even though we speculate that the observed associations between pan-fried meats and CRC risk are driven by formation of HCAs, in our study, the observed associations for the three considered individual HCAs were weak. Others and we have previously reported a similar discordance in other studies 49,53. Possible explanations include the fact that of the 17+ currently known HCAs that accumulate in meat, only three are captured by the CHARRED database; the possibility of misclassification in the estimation of HCAs, which would likely will lead to risk estimates that are biased toward the null; and lastly, lack of consideration of the fat and marinade used for pan-frying. Our fat and marinade questions referred to all red meats in general, without specifying the cooking method.

The second WCRF report 1,2, and a recent cohort study from Norway 39 support an association between total processed meats, ignoring cooking methods, and CRC risk. In our study, when ignoring cooking methods we only found evidence of an association for total processed meats and colon cancer, which was compatible with chance after a Bonferroni correction. Two other recent studies, reported no evidence of association between processed meats and CRC 24,54 and one study found a weak positive association 27.

Among the strengths of our study are the use of a population-based study that included close to 7000 individuals from North America, the use of a comprehensive FFQ, specifically designed to investigate the role of different meats, cooking practices, and HCAs; and consideration of possible heterogeneity of risk associations by tumor site and MMR status. Among the limitations of our study is the possibility of differential misclassification due to recall bias. However, some of the key associations we report are restricted to specific meat types, and specific cooking practices, not necessarily associated in the public media with stronger risk of CRC. Moreover, at the time of interview, cases were unaware of the MMR status of their tumor, therefore, recall bias could not explain our MMR-specific findings. We conducted many tests, and even though we applied a Bonferroni correction for the total number of meat variables considered (27), our approach might not be sufficient to account for false-positives. We chose not to correct further for subgroup analyses because we considered those only for associations that were statistically significant when considering CRC risk. However, we highlight that even if we applied a very stringent correction for all tests done (CRC and subgroup analyses) our main finding of an association with pan-fried beef would still remain statistically significant. Finally, as with all case–control studies of dietary variables, there is the possibility that both cases and controls may not have remembered their intake patterns accurately. This would lead to nondifferential misclassification and would bias ORs toward the null.

In summary, in a large population-based sample using a comprehensive FFQ, we found that frequent intake of pan-fried beefsteak and oven-broiled short ribs or spareribs may contribute to CRC risk, through a mechanism that may include HCA formation. Our findings suggest that, in addition to reducing the frequency of intake of these meat types, CRC risk can be lowered by taking precautions to reduce HCA formation in meats; this may be achieved by marinating meats in Asian style marinades, flipping meats often, reducing the pan temperature, and/or by preheating meats with microwave 55.

## Conflict of Interest

None declared.

## Supporting Information

**Table S1.** Meat intake and colorectal cancer risk, by cancer site and MSI status comparing cases to population-based controls. **Table S2.** Total red meat and poultry intake and CRC risk considering marinade use. **Table S3.** Heterocyclic amines and colorectal cancer risk, by cancer site and MMR status.
