# Supplementary material for: Meat intake, cooking methods, dietary carcinogens, and colorectal cancer risk: findings from the Colorectal Cancer Family Registry
Source: Cancer Med. 2015 Apr 7;4(6):936–52. doi: 10.1002/cam4.461 (PMC4472216; doi:10.1002/cam4.461)
Supplement: Supplementary file 1 [file cam40004-0936-sd1.pdf]

**Supplementary Table 1.** Meat intake and colorectal cancer risk, by cancer site and MSI status comparing cases to population-based controls

| | | | | Cancer site | | | | | | | MMR status | | | | | | |
|---|---|---|---|---|---|---|---|---|---|---|---|---|---|---|---|---|---|
| | Colorectal Cancer | | | Colon Cancer | | | Rectal Cancer | | | | MMR Proficient | | | MMR Deficient | | | |
| **Meat variables (g/1000Kcal/ day)** | Co/Ca | OR[1] | 95% CI | Cases | OR[1] | 95% CI | Cases | OR[1] | 95% CI | Het p | Cases | OR[1] | 95% CI | Cases | OR[1] | 95% CI | Het p |
| **Total non-processed Red Meat** | | | | | | | | | | | | | | | | | |
| Q1:0-10.8 | 330/633 | 1.0 REF | | 396 | 1.0 REF | | 151 | 1.0 REF | | | 171 | 1.0 REF | | 47 | 1.0 REF | | |
| Q2:10.81-16.04 | 351/644 | 1.0 | 0.8-1.3 | 380 | 1.0 | 0.8-1.3 | 168 | 0.9 | 0.7-1.3 | | 152 | 0.8 | 0.5-1.0 | 47 | 1.0 | 0.6-1.7 | |
| Q3:16.04-21.11 | 318/707 | 1.3 | 1.0-1.6 | 429 | 1.3 | 1.0-1.6 | 199 | 1.4 | 1.0-1.9 | | 201 | 1.1 | 0.8-1.5 | 53 | 1.2 | 0.7-2.1 | |
| Q4:21.12-28.19 | 309/680 | 1.3 | 1.0-1.6 | 396 | 1.2 | 0.9-1.5 | 187 | 1.2 | 0.8-1.6 | | 179 | 0.9 | 0.7-1.3 | 47 | 1.3 | 0.7-2.2 | |
| Q5:28.19-102.43 | 299/686 | 1.2 | 0.9-1.5 | 391 | 1.2 | 0.9-1.6 | 202 | 1.0 | 0.7-1.5 | | 173 | 0.8 | 0.6-1.2 | 49 | 1.2 | 0.7-2.1 | |
| p-for trend | | | 0.062 | | | 0.09 | | | 0.761 | 0.784 | | | 0.474 | | | 0.44 | 0.249 |
| **Beef** | | | | | | | | | | | | | | | | | |
| Q1:0-7.69 | 343/687 | 1.0 REF | | 436 | 1.0 REF | | 155 | 1.0 REF | | | 167 | 1.0 REF | | 42 | 1.0 REF | | |
| Q2:7.7-11.49 | 342/652 | 1.0 | 0.8-1.2 | 377 | 1.0 | 0.7-1.2 | 185 | 1.0 | 0.7-1.4 | | 165 | 0.9 | 0.7-1.3 | 54 | 1.2 | 0.7-2.1 | |
| Q3:11.5-15.08 | 329/654 | 1.1 | 0.9-1.3 | 396 | 1.0 | 0.8-1.3 | 174 | 1.1 | 0.8-1.6 | | 171 | 1.0 | 0.7-1.4 | 46 | 1.1 | 0.6-1.9 | |
| Q4:15.09-20.06 | 310/672 | 1.1 | 0.9-1.4 | 400 | 1.1 | 0.8-1.4 | 184 | 1.0 | 0.7-1.4 | | 192 | 1.0 | 0.7-1.3 | 55 | 1.5 | 0.9-2.6 | |
| Q5:20.08-76.3 | 283/685 | 1.2 | 0.9-1.5 | 383 | 1.1 | 0.9-1.5 | 209 | 1.2 | 0.8-1.6 | | 181 | 1.0 | 0.7-1.4 | 46 | 1.2 | 0.7-2.1 | |
| p-for trend | | | 0.159 | | | 0.264 | | | 0.422 | 0.284 | | | 0.947 | | | 0.516 | 0.76 |
| **Pork** | | | | | | | | | | | | | | | | | |
| Q1:0-1.32 | 363/617 | 1.0 REF | | 383 | 1.0 REF | | 154 | 1.0 REF | | | 159 | 1.0 REF | | 50 | 1.0 REF | | |
| Q2:1.33-3.01 | 319/641 | 1.2 | 0.9-1.5 | 388 | 1.2 | 0.9-1.5 | 163 | 1.1 | 0.8-1.6 | | 180 | 1.2 | 0.9-1.6 | 50 | 1.0 | 0.6-1.7 | |
| Q3:3.02-4.84 | 316/660 | 1.2 | 1.0-1.5 | 383 | 1.2 | 0.9-1.5 | 178 | 1.1 | 0.8-1.6 | | 160 | 0.9 | 0.7-1.3 | 49 | 1.1 | 0.7-1.9 | |
| Q4:4.85-7.43 | 293/743 | 1.5 | 1.2-1.9 | 440 | 1.5 | 1.1-1.9 | 207 | 1.5 | 1.1-2.1 | | 195 | 1.2 | 0.9-1.7 | 53 | 1.3 | 0.8-2.2 | |
| Q5:7.44-49.62 | 316/689 | 1.2 | 1.0-1.6 | 398 | 1.2 | 0.9-1.5 | 205 | 1.2 | 0.9-1.7 | | 182 | 1.0 | 0.7-1.4 | 41 | 1.0 | 0.6-1.7 | |
| p-for trend | | | 0.028 | | | 0.132 | | | 0.159 | 0.56 | | | 0.901 | | | 0.917 | 0.832 |

| | | | | | | | | | | | | | | | | | |
|---|---|---|---|---|---|---|---|---|---|---|---|---|---|---|---|---|---|
| **Organ Meat** | | | | | | | | | | | | | | | | | |
| Q1:0-0 | 514/884 | 1.0 REF | | 554 | 1.0 REF | | 206 | 1.0 REF | | | 250 | 1.0 REF | | 79 | 1.0 REF | | |
| Q2:0-0 | 139/282 | 1.1 | 0.9-1.5 | 152 | 1.1 | 0.8-1.5 | 91 | 1.3 | 0.9-1.9 | | 91 | 1.1 | 0.8-1.5 | 20 | 1.0 | 0.5-1.8 | |
| Q3:0-0 | 298/650 | 1.0 | 0.8-1.3 | 382 | 1.0 | 0.8-1.2 | 176 | 1.2 | 0.9-1.6 | | 178 | 1.0 | 0.8-1.4 | 49 | 1.1 | 0.7-1.8 | |
| Q4:0-.02 | 237/755 | 1.1 | 0.9-1.3 | 431 | 0.9 | 0.7-1.2 | 233 | 1.3 | 1.0-1.8 | | 181 | 0.9 | 0.7-1.3 | 38 | 0.9 | 0.5-1.5 | |
| Q5:.02-.64 | 419/779 | 1.1 | 0.9-1.3 | 473 | 1.1 | 0.9-1.4 | 201 | 1.1 | 0.8-1.5 | | 176 | 1.0 | 0.8-1.3 | 57 | 1.1 | 0.7-1.7 | |
| p-for trend | | | 0.555 | | | 0.324 | | | 0.75 | 0.22 | | | 0.965 | | | 0.654 | 0.204 |
| **Processed red meat** | | | | | | | | | | | | | | | | | |
| Q1:0-3.97 | 358/592 | 1.0 REF | | 347 | 1.0 REF | | 152 | 1.0 REF | | | 151 | 1.0 REF | | 46 | 1.0 REF | | |
| Q2:3.97-6.74 | 334/659 | 1.1 | 0.9-1.4 | 411 | 1.2 | 1.0-1.6 | 155 | 1.0 | 0.7-1.3 | | 154 | 1.0 | 0.7-1.3 | 64 | 1.3 | 0.8-2.1 | |
| Q3:6.75-9.53 | 311/618 | 1.0 | 0.8-1.3 | 368 | 1.1 | 0.9-1.4 | 165 | 1.0 | 0.7-1.3 | | 157 | 0.9 | 0.6-1.2 | 41 | 0.9 | 0.5-1.6 | |
| Q4:9.53-13.86 | 306/688 | 1.1 | 0.9-1.4 | 396 | 1.1 | 0.8-1.4 | 220 | 1.3 | 1.0-1.8 | | 202 | 1.2 | 0.8-1.6 | 39 | 0.8 | 0.4-1.4 | |
| Q5:13.87-105.25 | 298/793 | 1.2 | 0.9-1.5 | 470 | 1.3 | 1.0-1.7 | 215 | 1.0 | 0.7-1.5 | | 212 | 1.2 | 0.9-1.7 | 53 | 1.2 | 0.7-2.1 | |
| p-for trend | | | 0.189 | | | 0.229 | | | 0.46 | 0.74 | | | 0.077 | | | 0.801 | 0.326 |
| **Sausage & lunchmeats** | | | | | | | | | | | | | | | | | |
| Q1:0-.08 | 366/582 | 1.0 REF | | 350 | 1.0 REF | | 142 | 1.0 REF | | | 138 | 1.0 REF | | 44 | 1.0 REF | | |
| Q2:.08-.14 | 347/657 | 1.0 | 0.8-1.3 | 395 | 1.1 | 0.9-1.4 | 169 | 1.0 | 0.7-1.4 | | 148 | 0.9 | 0.7-1.3 | 58 | 1.1 | 0.6-1.7 | |
| Q3:.14-.22 | 290/706 | 1.4 | 1.1-1.7 | 409 | 1.5 | 1.1-1.9 | 190 | 1.2 | 0.9-1.7 | | 194 | 1.3 | 0.9-1.8 | 56 | 1.3 | 0.8-2.2 | |
| Q4:.22-.32 | 303/654 | 1.1 | 0.9-1.4 | 396 | 1.2 | 0.9-1.5 | 195 | 1.2 | 0.8-1.6 | | 179 | 1.1 | 0.8-1.6 | 40 | 0.7 | 0.4-1.3 | |
| Q5:.32-2.89 | 301/751 | 1.2 | 1.0-1.5 | 442 | 1.2 | 1.0-1.6 | 211 | 1.2 | 0.9-1.7 | | 217 | 1.4 | 1.0-1.9 | 45 | 1.1 | 0.6-1.8 | |
| p-for trend | | | 0.203 | | | 0.215 | | | 0.167 | 0.855 | | | 0.017 | | | 0.866 | 0.069 |
| **Poultry** | | | | | | | | | | | | | | | | | |
| Q1:0-7.86 | 378/634 | 1.0 REF | | 372 | 1.0 REF | | 183 | 1.0 REF | | | 173 | 1.0 REF | | 55 | 1.0 REF | | |
| Q2:7.86-11.85 | 355/642 | 1.0 | 0.8-1.2 | 384 | 1.1 | 0.8-1.4 | 166 | 0.9 | 0.7-1.2 | | 192 | 1.0 | 0.7-1.3 | 63 | 1.1 | 0.7-1.8 | |
| Q3:11.86-16.72 | 313/687 | 1.1 | 0.9-1.4 | 393 | 1.1 | 0.9-1.5 | 189 | 0.9 | 0.7-1.3 | | 183 | 1.1 | 0.8-1.5 | 49 | 0.9 | 0.5-1.4 | |
| Q4:16.73-24.66 | 288/664 | 1.0 | 0.8-1.3 | 396 | 1.1 | 0.8-1.4 | 180 | 0.9 | 0.7-1.2 | | 165 | 1.1 | 0.8-1.5 | 43 | 0.9 | 0.5-1.5 | |

| | | | | | | | | | | | | | | | | | |
|---|---|---|---|---|---|---|---|---|---|---|---|---|---|---|---|---|---|
| Q5:24.67-230.97 | 273/723 | 1.0 | 0.8-1.2 | 447 | 1.0 | 0.8-1.3 | 189 | 0.8 | 0.6-1.2 | | 163 | 1.1 | 0.8-1.5 | 33 | 0.6 | 0.4-1.1 | |
| p-for trend | | | 0.804 | | | 0.897 | | | 0.396 | 0.81 | | | 0.582 | | | 0.056 | 0.154 |
| | | | | | | | | | | | | | | | | | |
| **Processed poultry** | | | | | | | | | | | | | | | | | |
| Q1:0-.04 | 385/590 | 1.0 [REF] | | 353 | 1.0 [REF] | | 160 | 1.0 [REF] | | | 142 | 1.0 [REF] | | 43 | 1.0 [REF] | | |
| Q2:.04-.15 | 359/646 | 1.1 | 0.9-1.3 | 362 | 1.1 | 0.9-1.4 | 188 | 1.0 | 0.8-1.4 | | 161 | 1.0 | 0.8-1.4 | 60 | 1.7 | 1.1-2.8 | |
| Q3:.15-.63 | 310/698 | 1.2 | 1.0-1.5 | 407 | 1.2 | 1.0-1.6 | 186 | 1.1 | 0.8-1.5 | | 184 | 1.2 | 0.9-1.6 | 48 | 1.3 | 0.7-2.1 | |
| Q4:.63-1.65 | 265/657 | 1.1 | 0.9-1.4 | 395 | 1.2 | 0.9-1.5 | 166 | 1.1 | 0.8-1.5 | | 182 | 1.2 | 0.9-1.7 | 45 | 1.2 | 0.7-2.0 | |
| Q5:1.65-37.93 | 288/759 | 1.3 | 1.0-1.6 | 475 | 1.4 | 1.1-1.8 | 207 | 1.2 | 0.9-1.7 | | 207 | 1.5 | 1.1-2.0 | 47 | 1.3 | 0.8-2.2 | |
| p-for trend | | | 0.084 | | | 0.02 | | | 0.223 | 0.212 | | | 0.004 | | | 0.879 | 0.172 |
| | | | | | | | | | | | | | | | | | |
| **Total Processed Meats (Red Meat + Poultry)** | | | | | | | | | | | | | | | | | |
| Q1:0-4.43 | 357/593 | 1.0 [REF] | | 346 | 1.0 [REF] | | 155 | 1.0 [REF] | | | 146 | 1.0 [REF] | | 44 | 1.0 [REF] | | |
| Q2:4.43-7.35 | 342/643 | 1.0 | 0.8-1.3 | 395 | 1.2 | 0.9-1.5 | 159 | 0.9 | 0.6-1.2 | | 154 | 0.9 | 0.7-1.3 | 58 | 1.3 | 0.8-2.1 | |
| Q3:7.36-10.62 | 308/640 | 1.1 | 0.8-1.3 | 383 | 1.2 | 0.9-1.5 | 165 | 0.9 | 0.6-1.2 | | 161 | 0.9 | 0.6-1.2 | 53 | 1.2 | 0.7-2.0 | |
| Q4:10.63-15.29 | 315/654 | 1.0 | 0.8-1.3 | 385 | 1.0 | 0.8-1.4 | 200 | 1.1 | 0.8-1.6 | | 197 | 1.1 | 0.8-1.5 | 36 | 0.7 | 0.4-1.3 | |
| Q5:15.29-106.24 | 285/820 | 1.2 | 1.0-1.6 | 483 | 1.3 | 1.0-1.7 | 228 | 1.1 | 0.8-1.6 | | 218 | 1.3 | 1.0-1.8 | 52 | 1.2 | 0.7-2.2 | |
| p-for trend | | | 0.084 | | | 0.121 | | | 0.199 | 0.707 | | | 0.016 | | | 0.897 | 0.24 |
| | | | | | | | | | | | | | | | | | |
| **Pan Fried Beef Steak** | | | | | | | | | | | | | | | | | |
| Q1:0-0 | 940/1692 | 1.0 [REF] | | 1019 | 1.0 [REF] | | 449 | 1.0 [REF] | | | 469 | 1.0 [REF] | | 121 | 1.0 [REF] | | |
| Q2:.01-.02 | 215/506 | 1.0 | 0.8-1.2 | 300 | 1.0 | 0.8-1.3 | 146 | 0.8 | 0.6-1.2 | | 129 | 0.9 | 0.6-1.2 | 33 | 1.2 | 0.7-2.0 | |
| Q3:.02-.04 | 196/511 | 1.1 | 0.9-1.3 | 287 | 1.1 | 0.8-1.4 | 152 | 1.1 | 0.8-1.5 | | 119 | 0.9 | 0.7-1.2 | 35 | 1.1 | 0.6-1.9 | |
| Q4:.04-.99 | 231/619 | 1.2 | 1.0-1.5 | 374 | 1.4 | 1.1-1.7 | 152 | 1.0 | 0.7-1.3 | | 155 | 1.0 | 0.8-1.4 | 54 | 1.8 | 1.2-2.8 | |
| p-for trend | | | 0.054 | | | 0.011 | | | 0.85 | 0.38 | | | 0.939 | | | 0.01 | 0.062 |
| | | | | | | | | | | | | | | | | | |
| **Pan fried Hamburger** | | | | | | | | | | | | | | | | | |
| Q1:0-0 | 740/1297 | 1.0 [REF] | | 778 | 1.0 [REF] | | 352 | 1.0 [REF] | | | 381 | 1.0 [REF] | | 89 | 1.0 [REF] | | |
| Q2:.01-.02 | 258/627 | 1.1 | 0.9-1.4 | 369 | 1.1 | 0.9-1.4 | 169 | 1.0 | 0.7-1.3 | | 164 | 1.0 | 0.7-1.3 | 34 | 1.0 | 0.6-1.7 | |
| Q3:.02-.05 | 285/707 | 1.2 | 1.0-1.4 | 420 | 1.2 | 1.0-1.5 | 194 | 1.1 | 0.8-1.5 | | 178 | 1.0 | 0.7-1.3 | 56 | 1.7 | 1.1-2.6 | |

| | | | | | | | | | | | | | | | | | |
|---|---|---|---|---|---|---|---|---|---|---|---|---|---|---|---|---|---|
| Q4:.05-.99 | 302/697 | 1.1 | 0.9-1.4 | 410 | 1.1 | 0.9-1.4 | 186 | 1.1 | 0.9-1.5 | | 150 | 0.9 | 0.7-1.2 | 63 | 1.6 | 1.1-2.5 | |
| p-for trend | | | 0.122 | | | 0.179 | | | 0.295 | 0.515 | | | 0.532 | | | 0.01 | 0.028 |
| **Pan fried Chicken** | | | | | | | | | | | | | | | | | |
| Q1:0-0 | 728/1096 | 1.0 REF | | 656 | 1.0 REF | | 302 | 1.0 REF | | | 312 | 1.0 REF | | 98 | 1.0 REF | | |
| Q2:.01-.03 | 280/653 | 1.0 | 0.8-1.2 | 386 | 1.0 | 0.8-1.3 | 184 | 1.0 | 0.8-1.4 | | 194 | 1.1 | 0.8-1.4 | 40 | 0.6 | 0.4-1.1 | |
| Q3:.03-.07 | 295/781 | 1.2 | 1.0-1.5 | 453 | 1.3 | 1.0-1.6 | 215 | 1.2 | 1.0-1.6 | | 199 | 1.1 | 0.9-1.5 | 53 | 1.3 | 0.8-2.0 | |
| Q4:.07-1.18 | 286/788 | 1.1 | 0.9-1.4 | 475 | 1.1 | 0.9-1.4 | 197 | 1.1 | 0.8-1.4 | | 163 | 1.0 | 0.8-1.3 | 52 | 1.4 | 0.9-2.2 | |
| p-for trend | | | 0.105 | | | 0.152 | | | 0.479 | 0.94 | | | 0.81 | | | 0.034 | 0.159 |
| **Pan fried Sausage** | | | | | | | | | | | | | | | | | |
| Q1:0-0 | 749/1271 | 1.0 REF | | 789 | 1.0 REF | | 302 | 1.0 REF | | | 330 | 1.0 REF | | 101 | 1.0 REF | | |
| Q2:.01-.02 | 295/643 | 1.3 | 1.1-1.6 | 371 | 1.3 | 1.0-1.6 | 204 | 1.5 | 1.1-1.9 | | 205 | 1.4 | 1.1-1.8 | 46 | 1.2 | 0.7-1.8 | |
| Q3:.02-.04 | 298/619 | 1.1 | 0.9-1.3 | 356 | 1.0 | 0.8-1.3 | 177 | 1.4 | 1.0-1.8 | | 161 | 1.0 | 0.8-1.3 | 46 | 1.1 | 0.7-1.7 | |
| Q4:.04-1.32 | 253/781 | 1.2 | 1.0-1.5 | 456 | 1.2 | 0.9-1.5 | 213 | 1.4 | 1.0-1.9 | | 172 | 1.2 | 0.9-1.5 | 50 | 1.8 | 1.1-2.8 | |
| p-for trend | | | 0.053 | | | 0.246 | | | 0.027 | 0.054 | | | 0.517 | | | 0.014 | 0.523 |
| **Spam or Ham** | | | | | | | | | | | | | | | | | |
| Q1:0-0 | 1251/2097 | 1.0 REF | | 1240 | 1.0 REF | | 556 | 1.0 REF | | | 524 | 1.0 REF | | 173 | 1.0 REF | | |
| Q2:.01-.02 | 140/395 | 1.2 | 0.9-1.5 | 232 | 1.2 | 0.9-1.6 | 121 | 1.3 | 0.9-1.8 | | 106 | 1.6 | 1.1-2.2 | 18 | 0.5 | 0.3-1.1 | |
| Q3:.02-.04 | 104/403 | 1.2 | 0.9-1.6 | 245 | 1.3 | 0.9-1.7 | 109 | 1.3 | 0.9-2.0 | | 110 | 1.2 | 0.8-1.8 | 30 | 1.2 | 0.6-2.2 | |
| Q4:.04-.99 | 81/425 | 1.5 | 1.1-2.0 | 256 | 1.5 | 1.0-2.1 | 117 | 1.7 | 1.2-2.6 | | 128 | 2.1 | 1.5-3.1 | 19 | 0.6 | 0.3-1.5 | |
| p-for trend | | | 0.002 | | | 0.008 | | | 0.002 | 0.965 | | | <0.001 | | | 0.349 | 0.029 |
| **Pan fried Bacon** | | | | | | | | | | | | | | | | | |
| Q1:0-0 | 599/1094 | 1.0 REF | | 676 | 1.0 REF | | 268 | 1.0 REF | | | 292 | 1.0 REF | | 80 | 1.0 REF | | |
| Q2:.01-.03 | 325/664 | 1.0 | 0.8-1.2 | 374 | 1.0 | 0.8-1.2 | 212 | 1.2 | 0.9-1.6 | | 202 | 1.0 | 0.8-1.3 | 56 | 1.2 | 0.8-1.9 | |
| Q3:.03-.05 | 334/720 | 1.1 | 0.9-1.4 | 429 | 1.1 | 0.9-1.4 | 206 | 1.2 | 0.9-1.6 | | 216 | 1.1 | 0.9-1.5 | 54 | 1.2 | 0.7-1.9 | |
| Q4:.05-1.43 | 338/841 | 1.0 | 0.9-1.3 | 497 | 1.0 | 0.8-1.3 | 209 | 1.0 | 0.8-1.4 | | 160 | 0.9 | 0.6-1.2 | 53 | 1.1 | 0.7-1.8 | |
| p-for trend | | | 0.6 | | | 0.832 | | | 0.898 | 0.919 | | | 0.386 | | | 0.784 | 0.938 |

**Total Pan Fried Meat Intake**

| | | | | | | | | | | | | | | | | | |
|---|---|---|---|---|---|---|---|---|---|---|---|---|---|---|---|---|---|
| Q1:0-.05 | 442/638 | 1.0 REF | | 380 | 1.0 REF | | 168 | 1.0 REF | | | 195 | 1.0 REF | | 45 | 1.0 REF | | |
| Q2:.05-.12 | 406/739 | 1.2 | 1.0-1.4 | 434 | 1.2 | 0.9-1.5 | 216 | 1.2 | 0.9-1.6 | | 219 | 1.0 | 0.8-1.4 | 62 | 1.3 | 0.8-2.1 | |
| Q3:.12-.21 | 349/820 | 1.3 | 1.1-1.6 | 499 | 1.4 | 1.1-1.7 | 225 | 1.4 | 1.0-1.8 | | 224 | 1.0 | 0.8-1.4 | 61 | 1.7 | 1.1-2.8 | |
| Q4:.21-5.96 | 336/1051 | 1.3 | 1.1-1.6 | 622 | 1.3 | 1.0-1.6 | 266 | 1.2 | 0.9-1.7 | | 212 | 1.1 | 0.8-1.4 | 71 | 1.8 | 1.1-3.0 | |
| p-for trend | | | 0.016 | | | 0.038 | | | 0.221 | 0.91 | | | 0.632 | | | 0.015 | 0.075 |

**Oven-broiled Beef Steak**

| | | | | | | | | | | | | | | | | | |
|---|---|---|---|---|---|---|---|---|---|---|---|---|---|---|---|---|---|
| Q1:0-0 | 1108/2145 | 1.0 REF | | 1297 | 1.0 REF | | 555 | 1.0 REF | | | 569 | 1.0 REF | | 159 | 1.0 REF | | |
| Q2:.01-.02 | 137/399 | 1.2 | 0.9-1.5 | 226 | 1.1 | 0.9-1.5 | 128 | 1.3 | 0.9-1.8 | | 111 | 1.2 | 0.8-1.6 | 23 | 1.2 | 0.7-2.1 | |
| Q3:.02-.04 | 152/397 | 1.1 | 0.9-1.4 | 233 | 1.0 | 0.8-1.4 | 110 | 1.3 | 0.9-1.8 | | 104 | 1.1 | 0.8-1.5 | 22 | 1.0 | 0.6-1.7 | |
| Q4:.04-1.37 | 164/346 | 0.9 | 0.7-1.2 | 203 | 0.9 | 0.7-1.1 | 93 | 1.0 | 0.7-1.4 | | 76 | 1.0 | 0.7-1.4 | 36 | 1.2 | 0.7-2.0 | |
| p-for trend | | | 0.709 | | | 0.439 | | | 0.523 | 0.229 | | | 0.831 | | | 0.487 | 0.208 |

**Oven-broiled Hamburger**

| | | | | | | | | | | | | | | | | | |
|---|---|---|---|---|---|---|---|---|---|---|---|---|---|---|---|---|---|
| Q1:0-0 | 1250/2506 | 1.0 REF | | 1478 | 1.0 REF | | 679 | 1.0 REF | | | 688 | 1.0 REF | | 184 | 1.0 REF | | |
| Q2:.01-.02 | 106/241 | 0.8 | 0.6-1.1 | 134 | 0.8 | 0.6-1.1 | 75 | 0.9 | 0.6-1.3 | | 60 | 0.8 | 0.5-1.2 | 8 | 0.4 | 0.2-1.1 | |
| Q3:.02-.04 | 110/279 | 0.9 | 0.7-1.2 | 181 | 0.9 | 0.7-1.3 | 75 | 1.1 | 0.8-1.6 | | 55 | 0.9 | 0.6-1.3 | 19 | 1.0 | 0.6-1.9 | |
| Q4:.04-.99 | 111/283 | 1.0 | 0.8-1.3 | 178 | 1.0 | 0.7-1.4 | 65 | 0.9 | 0.6-1.4 | | 61 | 1.1 | 0.8-1.6 | 28 | 1.4 | 0.8-2.5 | |
| p-for trend | | | 0.758 | | | 0.749 | | | 0.878 | 0.712 | | | 0.916 | | | 0.321 | 0.327 |

**Oven-broiled Chicken**

| | | | | | | | | | | | | | | | | | |
|---|---|---|---|---|---|---|---|---|---|---|---|---|---|---|---|---|---|
| Q1:0-0 | 847/1585 | 1.0 REF | | 932 | 1.0 REF | | 425 | 1.0 REF | | | 408 | 1.0 REF | | 127 | 1.0 REF | | |
| Q2:.01-.03 | 231/568 | 1.0 | 0.8-1.3 | 335 | 1.1 | 0.8-1.4 | 175 | 1.0 | 0.8-1.4 | | 163 | 0.9 | 0.7-1.2 | 26 | 0.7 | 0.4-1.2 | |
| Q3:.03-.06 | 243/581 | 1.2 | 1.0-1.5 | 348 | 1.2 | 1.0-1.5 | 151 | 1.3 | 1.0-1.7 | | 164 | 1.3 | 1.0-1.6 | 42 | 1.0 | 0.6-1.6 | |
| Q4:.06-1.33 | 272/585 | 1.0 | 0.8-1.2 | 365 | 1.1 | 0.9-1.3 | 146 | 1.0 | 0.8-1.4 | | 133 | 1.0 | 0.8-1.3 | 46 | 1.2 | 0.8-1.9 | |
| p-for trend | | | 0.62 | | | 0.368 | | | 0.594 | 0.664 | | | 0.62 | | | 0.297 | 0.476 |

**Oven-broiled Shortribs or spareribs**

| | | | | | | | | | | | | | | | | | |
|---|---|---|---|---|---|---|---|---|---|---|---|---|---|---|---|---|---|
| Q1:0-0 | 1287/2389 | 1.0 REF | | 1423 | 1.0 REF | | 627 | 1.0 REF | | | 656 | 1.0 REF | | 178 | 1.0 REF | | |
| Q2:.01-.02 | 98/319 | 1.4 | 1.1-1.9 | 175 | 1.4 | 1.0-2.0 | 115 | 1.7 | 1.2-2.5 | | 91 | 1.5 | 1.1-2.2 | 15 | 1.1 | 0.6-2.2 | |
| Q3:.02-.03 | 96/299 | 1.4 | 1.1-1.9 | 189 | 1.4 | 1.0-1.9 | 74 | 1.3 | 0.9-2.0 | | 64 | 1.3 | 0.9-1.9 | 21 | 1.6 | 0.9-2.9 | |
| Q4:.03-.99 | 101/306 | 1.2 | 0.9-1.6 | 189 | 1.2 | 0.9-1.6 | 78 | 1.3 | 0.9-2.0 | | 58 | 1.1 | 0.8-1.7 | 26 | 1.9 | 1.1-3.4 | |
| p-for trend | | | 0.016 | | | 0.043 | | | 0.02 | 0.815 | | | 0.142 | | | 0.011 | 0.043 |
| **Total Oven Broiled Meat Intake** | | | | | | | | | | | | | | | | | |
| Q1:0-0 | 688/1237 | 1.0 REF | | 738 | 1.0 REF | | 320 | 1.0 REF | | | 335 | 1.0 REF | | 105 | 1.0 REF | | |
| Q2:.01-.05 | 274/664 | 1.1 | 0.9-1.3 | 376 | 1.1 | 0.9-1.4 | 202 | 1.2 | 0.9-1.5 | | 192 | 0.9 | 0.7-1.2 | 36 | 0.8 | 0.5-1.3 | |
| Q3:.05-.1 | 270/618 | 1.2 | 1.0-1.4 | 376 | 1.1 | 0.9-1.4 | 163 | 1.2 | 0.9-1.7 | | 161 | 1.1 | 0.8-1.4 | 38 | 0.8 | 0.5-1.4 | |
| Q4:.1-3.97 | 303/730 | 1.1 | 0.9-1.3 | 451 | 1.1 | 0.9-1.4 | 189 | 1.2 | 0.9-1.6 | | 162 | 1.1 | 0.8-1.4 | 57 | 1.2 | 0.8-1.8 | |
| p-for trend | | | 0.497 | | | 0.469 | | | 0.233 | 0.796 | | | 0.463 | | | 0.41 | 0.269 |
| **Grilled Beef Steak** | | | | | | | | | | | | | | | | | |
| Q1:0-0 | 554/1314 | 1.0 REF | | 814 | 1.0 REF | | 295 | 1.0 REF | | | 281 | 1.0 REF | | 78 | 1.0 REF | | |
| Q2:.01-.02 | 310/726 | 1.0 | 0.8-1.2 | 413 | 0.9 | 0.8-1.2 | 243 | 1.2 | 0.9-1.6 | | 200 | 0.9 | 0.6-1.1 | 53 | 1.1 | 0.7-1.8 | |
| Q3:.02-.04 | 356/677 | 0.9 | 0.8-1.1 | 401 | 0.9 | 0.8-1.2 | 187 | 1.0 | 0.8-1.3 | | 201 | 0.9 | 0.7-1.2 | 56 | 1.2 | 0.7-1.8 | |
| Q4:.04-.99 | 355/582 | 0.9 | 0.7-1.0 | 335 | 0.8 | 0.7-1.0 | 166 | 0.9 | 0.7-1.2 | | 177 | 0.8 | 0.6-1.1 | 55 | 1.3 | 0.8-2.0 | |
| p-for trend | | | 0.12 | | | 0.099 | | | 0.423 | 0.475 | | | 0.227 | | | 0.274 | 0.353 |
| **Grilled Hamburger** | | | | | | | | | | | | | | | | | |
| Q1:0-0 | 544/1401 | 1.0 REF | | 844 | 1.0 REF | | 332 | 1.0 REF | | | 336 | 1.0 REF | | 81 | 1.0 REF | | |
| Q2:.01-.02 | 313/690 | 0.9 | 0.8-1.1 | 399 | 1.0 | 0.8-1.2 | 219 | 1.1 | 0.8-1.5 | | 167 | 0.7 | 0.6-1.0 | 47 | 1.0 | 0.6-1.6 | |
| Q3:.02-.05 | 376/686 | 0.9 | 0.7-1.1 | 407 | 0.9 | 0.7-1.1 | 198 | 1.0 | 0.8-1.4 | | 207 | 0.8 | 0.6-1.1 | 61 | 1.1 | 0.7-1.7 | |
| Q4:.05-.99 | 352/542 | 0.8 | 0.6-0.9 | 326 | 0.8 | 0.6-1.0 | 149 | 0.8 | 0.6-1.1 | | 157 | 0.7 | 0.6-1.0 | 53 | 1.2 | 0.7-1.8 | |
| p-for trend | | | 0.007 | | | 0.062 | | | 0.09 | 0.801 | | | 0.075 | | | 0.493 | 0.27 |
| **Grilled Chicken** | | | | | | | | | | | | | | | | | |
| Q1:0-0 | 562/1195 | 1.0 REF | | 729 | 1.0 REF | | 280 | 1.0 REF | | | 251 | 1.0 REF | | 84 | 1.0 REF | | |
| Q2:.01-.03 | 314/835 | 1.0 | 0.9-1.3 | 482 | 1.1 | 0.8-1.3 | 254 | 1.2 | 0.9-1.5 | | 232 | 1.2 | 0.9-1.5 | 58 | 1.3 | 0.8-2.1 | |

| | | | | | | | | | | | | | | | | | |
|---|---|---|---|---|---|---|---|---|---|---|---|---|---|---|---|---|---|
| Q3:.03-.06 | 361/691 | 0.9 | 0.8-1.1 | 408 | 0.9 | 0.7-1.2 | 200 | 1.0 | 0.7-1.3 | | 200 | 0.9 | 0.7-1.2 | 58 | 1.3 | 0.8-2.0 | |
| Q4:.06-1.15 | 352/604 | 0.9 | 0.7-1.1 | 360 | 0.9 | 0.7-1.1 | 168 | 0.9 | 0.7-1.2 | | 188 | 1.1 | 0.8-1.4 | 43 | 0.9 | 0.6-1.5 | |
| p-for trend | | | 0.136 | | | 0.205 | | | 0.251 | 0.346 | | | 0.772 | | | 0.712 | 0.252 |
| **Grilled Sausage** | | | | | | | | | | | | | | | | | |
| Q1:0-0 | 1005/2222 | 1.0 REF | | 1321 | 1.0 REF | | 571 | 1.0 REF | | | 507 | 1.0 REF | | 162 | 1.0 REF | | |
| Q2:.01-.02 | 187/410 | 1.2 | 1.0-1.6 | 237 | 1.3 | 1.0-1.6 | 134 | 1.3 | 1.0-1.8 | | 147 | 1.4 | 1.1-1.9 | 28 | 1.1 | 0.6-1.8 | |
| Q3:.02-.03 | 177/327 | 1.0 | 0.8-1.3 | 208 | 1.1 | 0.9-1.4 | 88 | 0.9 | 0.7-1.3 | | 111 | 1.2 | 0.9-1.6 | 21 | 1.0 | 0.6-1.6 | |
| Q4:.03-.99 | 214/357 | 1.0 | 0.8-1.2 | 209 | 1.0 | 0.8-1.2 | 105 | 1.0 | 0.7-1.4 | | 104 | 1.1 | 0.8-1.5 | 32 | 1.4 | 0.9-2.1 | |
| p-for trend | | | 0.797 | | | 0.977 | | | 0.981 | 0.924 | | | 0.379 | | | 0.229 | 0.68 |
| **Grilled Shortribs or spareribs** | | | | | | | | | | | | | | | | | |
| Q1:0-0 | 1175/2239 | 1.0 REF | | 1341 | 1.0 REF | | 575 | 1.0 REF | | | 602 | 1.0 REF | | 173 | 1.0 REF | | |
| Q2:.01-.02 | 137/360 | 1.0 | 0.7-1.2 | 205 | 1.0 | 0.7-1.3 | 117 | 1.0 | 0.7-1.4 | | 101 | 1.0 | 0.7-1.4 | 19 | 0.7 | 0.4-1.4 | |
| Q3:.02-.03 | 130/344 | 1.1 | 0.9-1.4 | 203 | 1.1 | 0.9-1.5 | 111 | 1.4 | 1.0-1.9 | | 79 | 1.1 | 0.8-1.5 | 23 | 1.3 | 0.7-2.2 | |
| Q4:.03-.99 | 134/361 | 1.0 | 0.8-1.3 | 221 | 1.0 | 0.8-1.4 | 88 | 1.0 | 0.7-1.4 | | 83 | 1.0 | 0.7-1.4 | 26 | 1.3 | 0.8-2.2 | |
| p-for trend | | | 0.761 | | | 0.595 | | | 0.661 | 0.934 | | | 1 | | | 0.28 | 0.411 |
| **Total Grilled Meat Intake** | | | | | | | | | | | | | | | | | |
| Q1:0-0 | 315/736 | 1.0 REF | | 456 | 1.0 REF | | 159 | 1.0 REF | | | 157 | 1.0 REF | | 46 | 1.0 REF | | |
| Q2:.01-.07 | 363/944 | 1.0 | 0.8-1.3 | 551 | 1.0 | 0.8-1.3 | 273 | 1.2 | 0.9-1.6 | | 245 | 0.8 | 0.6-1.1 | 65 | 1.1 | 0.6-1.8 | |
| Q3:.07-.15 | 426/805 | 0.9 | 0.7-1.1 | 463 | 0.8 | 0.6-1.0 | 238 | 1.1 | 0.8-1.4 | | 220 | 0.7 | 0.5-1.0 | 68 | 1.0 | 0.6-1.6 | |
| Q4:.15-4.97 | 430/757 | 0.9 | 0.7-1.1 | 465 | 0.9 | 0.7-1.2 | 202 | 0.9 | 0.6-1.2 | | 224 | 0.8 | 0.6-1.1 | 60 | 1.1 | 0.7-1.8 | |
| p-for trend | | | 0.122 | | | 0.358 | | | 0.084 | 0.998 | | | 0.336 | | | 0.667 | 0.859 |
| **PhIP** | | | | | | | | | | | | | | | | | |
| Q1:0-60.61 | 363/685 | 1.0 REF | | 406 | 1.0 REF | | 181 | 1.0 REF | | | 184 | 1.0 REF | | 50 | 1.0 REF | | |
| Q2:60.68-112.61 | 318/634 | 0.9 | 0.8-1.2 | 368 | 0.9 | 0.7-1.2 | 190 | 1.0 | 0.7-1.3 | | 168 | 0.8 | 0.6-1.1 | 43 | 0.9 | 0.5-1.6 | |
| Q3:112.65-185.26 | 316/654 | 0.9 | 0.7-1.1 | 386 | 1.0 | 0.8-1.3 | 176 | 0.8 | 0.6-1.0 | | 170 | 0.8 | 0.6-1.1 | 45 | 1.2 | 0.7-2.0 | |
| Q4:185.35-315.04 | 304/639 | 0.9 | 0.8-1.2 | 373 | 1.0 | 0.7-1.2 | 183 | 0.9 | 0.7-1.2 | | 174 | 0.9 | 0.6-1.2 | 49 | 1.3 | 0.8-2.2 | |

| | | | | | | | | | | | | | | | | | |
|---|---|---|---|---|---|---|---|---|---|---|---|---|---|---|---|---|---|
| Q5:315.12-4922.97 | 306/738 | 0.9 | 0.7-1.1 | 459 | 1.0 | 0.8-1.3 | 177 | 0.7 | 0.5-1.0 | | 180 | 0.8 | 0.6-1.1 | 56 | 1.5 | 0.9-2.5 | |
| p-for trend | | | 0.362 | | | 0.843 | | | 0.033 | 0.162 | | | 0.504 | | | 0.036 | 0.223 |
| **DMeIQx** | | | | | | | | | | | | | | | | | |
| Q1:0-11.83 | 354/631 | 1.0 REF | | 364 | 1.0 REF | | 179 | 1.0 REF | | | 194 | 1.0 REF | | 40 | 1.0 REF | | |
| Q2:11.84-23.43 | 309/635 | 1.1 | 0.9-1.3 | 380 | 1.2 | 0.9-1.5 | 176 | 0.9 | 0.7-1.2 | | 167 | 0.7 | 0.6-1.0 | 45 | 1.6 | 0.9-2.8 | |
| Q3:23.48-40.18 | 301/635 | 1.1 | 0.8-1.3 | 374 | 1.1 | 0.9-1.4 | 184 | 0.9 | 0.7-1.3 | | 176 | 0.8 | 0.6-1.1 | 45 | 1.5 | 0.8-2.7 | |
| Q4:40.21-69.93 | 336/676 | 1.0 | 0.8-1.2 | 409 | 1.1 | 0.8-1.4 | 173 | 0.7 | 0.5-1.0 | | 155 | 0.7 | 0.5-0.9 | 55 | 1.7 | 1.0-3.0 | |
| Q5:70.03-1188.52 | 307/773 | 1.0 | 0.8-1.3 | 465 | 1.1 | 0.8-1.4 | 195 | 0.9 | 0.6-1.2 | | 184 | 0.8 | 0.6-1.1 | 58 | 2.1 | 1.2-3.6 | |
| p-for trend | | | 0.752 | | | 0.85 | | | 0.326 | 0.335 | | | 0.559 | | | 0.019 | 0.145 |
| **DDiMeIQx** | | | | | | | | | | | | | | | | | |
| Q1:0-.82 | 336/713 | 1.0 REF | | 431 | 1.0 REF | | 180 | 1.0 REF | | | 191 | 1.0 REF | | 50 | 1.0 REF | | |
| Q2:.82-1.65 | 320/641 | 0.9 | 0.7-1.1 | 365 | 0.9 | 0.7-1.2 | 197 | 0.8 | 0.6-1.1 | | 178 | 0.8 | 0.6-1.1 | 35 | 0.9 | 0.5-1.5 | |
| Q3:1.65-2.62 | 317/570 | 0.8 | 0.7-1.0 | 344 | 0.9 | 0.7-1.1 | 152 | 0.7 | 0.5-1.0 | | 144 | 0.7 | 0.5-0.9 | 42 | 1.2 | 0.7-2.1 | |
| Q4:2.62-4.71 | 317/707 | 0.9 | 0.8-1.2 | 418 | 1.0 | 0.8-1.3 | 202 | 0.9 | 0.7-1.3 | | 178 | 0.8 | 0.6-1.1 | 56 | 1.4 | 0.8-2.4 | |
| Q5:4.71-173.75 | 317/719 | 0.9 | 0.7-1.1 | 434 | 1.0 | 0.7-1.2 | 176 | 0.7 | 0.5-1.0 | | 185 | 0.9 | 0.6-1.2 | 60 | 1.6 | 0.9-2.6 | |
| p-for trend | | | 0.39 | | | 0.994 | | | 0.09 | 0.642 | | | 0.689 | | | 0.019 | 0.175 |
| **Total heterocyclic amines** | | | | | | | | | | | | | | | | | |
| Q1:0-82.04 | 357/698 | 1.0 REF | | 402 | 1.0 REF | | 196 | 1.0 REF | | | 190 | 1.0 REF | | 49 | 1.0 REF | | |
| Q2:82.12-140.75 | 321/573 | 0.9 | 0.7-1.1 | 344 | 0.9 | 0.7-1.2 | 165 | 0.8 | 0.6-1.1 | | 158 | 0.8 | 0.6-1.0 | 49 | 0.9 | 0.5-1.6 | |
| Q3:140.82-227.37 | 311/664 | 0.9 | 0.7-1.1 | 385 | 1.0 | 0.8-1.2 | 179 | 0.7 | 0.5-1.0 | | 165 | 0.8 | 0.6-1.1 | 39 | 0.9 | 0.5-1.6 | |
| Q4:227.46-387.49 | 305/680 | 1.0 | 0.8-1.2 | 415 | 1.1 | 0.8-1.4 | 185 | 0.9 | 0.6-1.2 | | 188 | 0.9 | 0.7-1.2 | 51 | 1.4 | 0.9-2.3 | |
| Q5:387.97-6166.49 | 313/735 | 0.8 | 0.7-1.0 | 446 | 0.9 | 0.7-1.2 | 182 | 0.7 | 0.5-0.9 | | 175 | 0.8 | 0.6-1.1 | 55 | 1.3 | 0.8-2.2 | |
| p-for trend | | | 0.253 | | | 0.827 | | | 0.036 | 0.249 | | | 0.362 | | | 0.099 | 0.425 |

OR[1] Adjusted for age (years, continuous), BMI (<25, 25.0-29.9, ≥30), gender, race (NHW, AA, Asian and others), saturated fat (g/ 1000 kcal/ day), dietary fiber (g/ 1000 kcal/ day), center, vegetables (g/ 1000 Kcal/ day), physical activity (hrs/ week, continuous) and total calorie intake (kcal/day, continuous).

**Supplementary Table 2.** Total red meat and poultry intake and CRC risk considering marinade use

| Meat variables (g/1000Kcal/ day) | CO/CA | OR[1] | 95% CI | Cases | OR[1] | 95% CI | Cases | OR[1] | 95% CI |
|---|---|---|---|---|---|---|---|---|---|
| | **No BBQ** | | | **Low BBQ** | | | **High BBQ** | | |
| **Non-Processed Red-Meat** | | | | | | | | | |
| Q1:0-10.8 | 549/465 | 1.0 REF | | 95/106 | 1.0 REF | | 55/58 | 1.0 REF | |
| Q2:10.81-16.04 | 405/397 | 1.2 | 1.0-1.5 | 167/149 | 0.8 | 0.5-1.1 | 124/95 | 0.8 | 0.5-1.4 |
| Q3:16.04-21.11 | 369/379 | 1.3 | 1.1-1.6 | 177/180 | 0.9 | 0.6-1.3 | 145/146 | 1.2 | 0.8-2.0 |
| Q4:21.12-28.19 | 345/344 | 1.3 | 1.1-1.7 | 183/161 | 0.8 | 0.6-1.2 | 164/172 | 1.2 | 0.7-2.0 |
| Q5:28.19-102.43 | 359/321 | 1.2 | 0.9-1.4 | 173/155 | 0.8 | 0.5-1.1 | 167/207 | 1.5 | 0.9-2.4 |
| p-for trend | | | 0.173 | | | 0.268 | | | 0.01 |
| p for heterogeneity | 0.084 | | | | | | | | |
| **Non-Processed Poultry** | | | | | | | | | |
| Q1:0-7.86 | 532/459 | 1.0 REF | | 95/101 | 1.0 REF | | 71/70 | 1.0 REF | |
| Q2:7.86-11.85 | 454/411 | 1.1 | 0.9-1.3 | 137/101 | 0.7 | 0.5-1.1 | 97/124 | 1.3 | 0.8-2.0 |
| Q3:11.86-16.72 | 410/370 | 1.1 | 0.9-1.3 | 154/153 | 0.9 | 0.6-1.4 | 133/156 | 1.1 | 0.7-1.7 |
| Q4:16.73-24.66 | 388/363 | 1.1 | 0.9-1.3 | 147/152 | 1.0 | 0.7-1.4 | 155/143 | 0.8 | 0.5-1.2 |
| Q5:24.67-230.97 | 381/383 | 1.0 | 0.8-1.2 | 169/155 | 0.7 | 0.5-1.0 | 141/182 | 1.1 | 0.7-1.6 |
| P for trend | | | 0.959 | | | 0.162 | | | 0.585 |
| p for heterogeneity | 0.131 | | | | | | | | |
| | **No Shoyu** | | | **Low Shoyu** | | | **High Shoyu** | | |
| **Non-Processed Red-Meat** | | | | | | | | | |
| Q1:0-10.8 | 560/475 | 1.0 REF | | 95/96 | 1.0 REF | | 39/59 | 1.0 REF | |
| Q2:10.81-16.04 | 459/450 | 1.2 | 1.0-1.5 | 125/117 | 1.0 | 0.7-1.6 | 105/70 | 0.4 | 0.2-0.7 |
| Q3:16.04-21.11 | 423/466 | 1.5 | 1.2-1.8 | 160/125 | 0.8 | 0.5-1.2 | 102/111 | 0.7 | 0.4-1.1 |
| Q4:21.12-28.19 | 401/418 | 1.4 | 1.2-1.7 | 149/116 | 0.9 | 0.6-1.3 | 138/140 | 0.6 | 0.4-1.0 |
| Q5:28.19-102.43 | 407/406 | 1.3 | 1.1-1.6 | 132/107 | 0.8 | 0.5-1.2 | 155/170 | 0.6 | 0.4-1.1 |
| p-for trend | | | 0.007 | | | 0.189 | | | 0.979 |
| P for heterogeneity | 0.008 | | | | | | | | |
| **Non-Processed Poultry** | | | | | | | | | |
| Q1:0-7.86 | 561/504 | 1.0 REF | | 87/70 | 1.0 REF | | 43/52 | 1.0 REF | |
| Q2:7.86-11.85 | 475/457 | 1.1 | 0.9-1.3 | 126/91 | 0.9 | 0.6-1.4 | 84/86 | 0.8 | 0.5-1.3 |
| Q3:11.86-16.72 | 440/441 | 1.1 | 0.9-1.3 | 120/117 | 1.3 | 0.8-2.0 | 123/117 | 0.8 | 0.5-1.3 |
| Q4:16.73-24.66 | 419/399 | 1.0 | 0.8-1.2 | 126/123 | 1.3 | 0.8-1.9 | 137/136 | 0.8 | 0.5-1.3 |
| Q5:24.67-230.97 | 397/455 | 1.1 | 0.9-1.3 | 138/104 | 0.9 | 0.6-1.4 | 155/160 | 0.7 | 0.4-1.2 |
| p-for trend | | | 0.857 | | | 0.64 | | | 0.282 |
| P for heterogeneity | 0.551 | | | | | | | | |

OR[1] Adjusted for age (years, continuous), BMI (<25, 25.0-29.9, ≥30), gender, race (NHW, AA, Asian and others), saturated fat (g/ 1000 kcal/ day), dietary fiber (g/ 1000 kcal/ day), center, vegetables (g/ 1000 Kcal/ day), physical activity (hrs/ week, continuous) and total calorie intake (kcal/day, continuous).

**Supplementary Table 3.** Heterocyclic amines and colorectal cancer risk, by cancer site an[illegible]

| | | | | Cancer site | | | | | | | MMR status | | | | | | |
|---|---|---|---|---|---|---|---|---|---|---|---|---|---|---|---|---|---|
| | Colorectal Cancer | | | Colon Cancer | | | Rectal Cancer | | | | MMR Proficient | | | MMR Deficient | | | |
| **g/1000Kcal/ day** | CO/CA | OR[1] | 95% CI | Cases | OR[1] | 95% CI | Cases | OR[1] | 95% CI | Het p | Cases | OR[1] | 95% CI | Cases | OR[1] | 95% CI | Het p |
| **PhIP** | | | | | | | | | | | | | | | | | |
| Q1 | 702/685 | 1.0 REF | | 406 | 1.0 REF | | 181 | 1.0 REF | | | 184 | 1.0 REF | | 50 | 1.0 REF | | |
| Q2 | 701/634 | 0.9 | 0.8-1.1 | 368 | 0.9 | 0.7-1.1 | 190 | 1 | 0.8-1.2 | | 168 | 0.9 | 0.7-1.1 | 43 | 0.9 | 0.6-1.3 | |
| Q3 | 701/654 | 0.9 | 0.8-1.1 | 386 | 1.0 | 0.8-1.2 | 176 | 0.9 | 0.7-1.1 | | 170 | 0.8 | 0.7-1.1 | 45 | 0.9 | 0.6-1.4 | |
| Q4 | 700/639 | 0.9 | 0.8-1.1 | 373 | 0.9 | 0.8-1.1 | 183 | 0.9 | 0.7-1.2 | | 174 | 0.9 | 0.7-1.1 | 49 | 1.0 | 0.7-1.6 | |
| Q5 | 700/738 | 0.9 | 0.8-1.1 | 459 | 1.0 | 0.8-1.2 | 177 | 0.9 | 0.7-1.1 | | 180 | 1.0 | 0.8-1.2 | 56 | 1.3 | 0.8-1.9 | |
| p-for trend | | | 0.67 | | | 0.67 | | | 0.216 | 0.158 | | | 0.733 | | | 0.113 | 0.23 |
| **DMeIQx** | | | | | | | | | | | | | | | | | |
| Q1 | 702/631 | 1.0 REF | | 364 | 1.0 REF | | 179 | 1.0 REF | | | 194 | 1.0 REF | | 40 | 1.0 REF | | |
| Q2 | 701/635 | 1 | 0.8-1.1 | 380 | 1.0 | 0.8-1.2 | 176 | 0.9 | 0.7-1.1 | | 167 | 0.8 | 0.6-1.0 | 45 | 1.1 | 0.7-1.7 | |
| Q3 | 701/635 | 0.9 | 0.8-1.1 | 374 | 1.0 | 0.8-1.2 | 184 | 0.9 | 0.7-1.1 | | 176 | 0.8 | 0.6-1.0 | 45 | 1.1 | 0.7-1.7 | |
| Q4 | 700/676 | 0.9 | 0.8-1.1 | 409 | 1.0 | 0.8-1.2 | 173 | 0.8 | 0.6-1.0 | | 155 | 0.7 | 0.6-0.9 | 55 | 1.3 | 0.8-2.0 | |
| Q5 | 700/773 | 1.0 | 0.9-1.2 | 465 | 1.1 | 0.9-1.3 | 195 | 0.9 | 0.7-1.2 | | 184 | 0.9 | 0.7-1.2 | 58 | 1.4 | 0.9-2.2 | |
| p-for trend | | | 0.59 | | | 0.305 | | | 0.796 | 0.335 | | | 0.954 | | | 0.070 | 0.14 |
| **DDiMeIQx** | | | | | | | | | | | | | | | | | |
| Q1 | 702/713 | 1.0 REF | | 431 | 1.0 REF | | 180 | 1.0 REF | | | 191 | 1.0 REF | | 50 | 1.0 REF | | |
| Q2 | 701/641 | 0.9 | 0.8-1.1 | 365 | 0.9 | 0.7-1.1 | 197 | 1.0 | 0.8-1.3 | | 178 | 0.8 | 0.7-1.1 | 35 | 0.7 | 0.4-1.0 | |
| Q3 | 701/570 | 0.8 | 0.7-0.9 | 344 | 0.8 | 0.7-1.0 | 152 | 0.8 | 0.6-1.0 | | 144 | 0.7 | 0.5-0.9 | 42 | 0.9 | 0.6-1.3 | |
| Q4 | 700/707 | 1.0 | 0.8-1.1 | 418 | 1.0 | 0.8-1.2 | 202 | 1.0 | 0.8-1.3 | | 178 | 0.9 | 0.7-1.1 | 56 | 1.1 | 0.7-1.6 | |
| Q5 | 700/719 | 0.9 | 0.8-1.1 | 434 | 1.0 | 0.8-1.2 | 176 | 0.9 | 0.7-1.2 | | 185 | 1.0 | 0.8-1.2 | 60 | 1.2 | 0.8-1.8 | |
| p-for trend | | | 0.956 | | | 0.614 | | | 0.724 | 0.64 | | | 0.448 | | | 0.042 | 0.17 |
| **Total heterocyclic amines** | | | | | | | | | | | | | | | | | |
| Q1 | 702/698 | 1.0 REF | | 402 | 1.0 REF | | 196 | 1.0 REF | | | 190 | 1.0 REF | | 49 | 1.0 REF | | |
| Q2 | 701/573 | 0.8 | 0.7-1.0 | 344 | 0.9 | 0.7-1.0 | 165 | 0.8 | 0.6-1.0 | | 158 | 0.8 | 0.6-1.0 | 49 | 1.0 | 0.7-1.6 | |
| Q3 | 701/664 | 0.9 | 0.8-1.1 | 385 | 0.9 | 0.8-1.1 | 179 | 0.8 | 0.7-1.0 | | 165 | 0.8 | 0.6-1.0 | 39 | 0.8 | 0.5-1.3 | |

| | | | | | | | | | | | | | | | | | |
|---|---|---|---|---|---|---|---|---|---|---|---|---|---|---|---|---|---|
| Q4 | 700/680 | 0.9 | 0.8-1.1 | 415 | 1.0 | 0.8-1.2 | 185 | 0.8 | 0.7-1.1 | | 188 | 0.9 | 0.7-1.2 | 51 | 1.1 | 0.7-1.7 | |
| Q5 | 700/735 | 0.9 | 0.8-1.1 | 446 | 1.0 | 0.8-1.2 | 182 | 0.8 | 0.6-1.0 | | 175 | 0.9 | 0.7-1.2 | 55 | 1.2 | 0.8-1.8 | |
| p-for trend | | | 0.776 | | | 0.725 | | | 0.332 | 0.248 | | | 0.763 | | | 0.203 | 0.43 |

OR[1] Adjusted for age (years, continuous), BMI (<25, 25.0-29.9, ≥30), gender, race (NHW, AA, Asian and others), saturated fat (g/ 1000 kcal/ day),
